# Dementia Revealed: Novel Chromosome 6 Locus for Late-Onset Alzheimer Disease Provides Genetic Evidence for Folate-Pathway Abnormalities

**DOI:** 10.1371/journal.pgen.1001130

**Published:** 2010-09-23

**Authors:** Adam C. Naj, Gary W. Beecham, Eden R. Martin, Paul J. Gallins, Eric H. Powell, Ioanna Konidari, Patrice L. Whitehead, Guiqing Cai, Vahram Haroutunian, William K. Scott, Jeffery M. Vance, Michael A. Slifer, Harry E. Gwirtsman, John R. Gilbert, Jonathan L. Haines, Joseph D. Buxbaum, Margaret A. Pericak-Vance

**Affiliations:** 1 Dr. John T. Macdonald Foundation Department of Human Genetics, John P. Hussman Institute for Human Genomics, University of Miami Miller School of Medicine, Miami, Florida, United States of America; 2 Department of Psychiatry, Mount Sinai School of Medicine, New York, New York, United States of America; 3 Veterans Affairs Medical Center, Nashville, Tennessee, United States of America; 4 Vanderbilt Center for Human Genetics Research, Vanderbilt University, Nashville, Tennessee, United States of America; University of Washington, United States of America

## Abstract

Genome-wide association studies (GWAS) of late-onset Alzheimer disease (LOAD) have consistently observed strong evidence of association with polymorphisms in *APOE*. However, until recently, variants at few other loci with statistically significant associations have replicated across studies. The present study combines data on 483,399 single nucleotide polymorphisms (SNPs) from a previously reported GWAS of 492 LOAD cases and 496 controls and from an independent set of 439 LOAD cases and 608 controls to strengthen power to identify novel genetic association signals. Associations exceeding the experiment-wide significance threshold (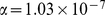) were replicated in an additional 1,338 cases and 2,003 controls. As expected, these analyses unequivocally confirmed *APOE*'s risk effect (rs2075650, 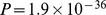). Additionally, the SNP rs11754661 at 151.2 Mb of chromosome 6q25.1 in the gene *MTHFD1L* (which encodes the methylenetetrahydrofolate dehydrogenase (NADP+ dependent) 1-like protein) was significantly associated with LOAD (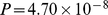; Bonferroni-corrected *P* = 0.022). Subsequent genotyping of SNPs in high linkage disequilibrium (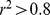) with rs11754661 identified statistically significant associations in multiple SNPs (rs803424, *P* = 0.016; rs2073067, *P* = 0.03; rs2072064, *P* = 0.035), reducing the likelihood of association due to genotyping error. In the replication case-control set, we observed an association of rs11754661 in the same direction as the previous association at *P* = 0.002 (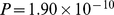 in combined analysis of discovery and replication sets), with associations of similar statistical significance at several adjacent SNPs (rs17349743, *P* = 0.005; rs803422, *P* = 0.004). In summary, we observed and replicated a novel statistically significant association in *MTHFD1L*, a gene involved in the tetrahydrofolate synthesis pathway. This finding is noteworthy, as *MTHFD1L* may play a role in the generation of methionine from homocysteine and influence homocysteine-related pathways and as levels of homocysteine are a significant risk factor for LOAD development.

## Author Summary

Studies looking for genetic variants across the genome that affect late-onset Alzheimer disease (LOAD) have had little success identifying genes other than *APOE*. Here, we use an expanded set of AD cases and controls to improve our power to detect genetic variants driving LOAD risk. Analyzing 483,399 genetic variants across the genome in a discovery dataset of 931 cases and 1,104 controls, we found a strong association to the marker rs11754661 on chromosome 6 in the gene *MTHFD1L*, in addition to the highly replicated chromosome 19 *APOE* association. We genotyped adjacent variants on chromosome 6 in these same cases and controls and found these variants were also associated with LOAD. We replicated the association with rs11754661 and additional SNPs in *MTHFD1L* in a combined dataset of cases and controls from our laboratory and from publicly available datasets. This finding is important because the gene is known to be involved in biological pathways influencing levels of homocysteine, a significant risk factor for AD.

## Introduction

Alzheimer disease (AD) [MIM 104300] is a neurodegenerative disorder characterized by memory and cognitive impairment affecting more than 13% of individuals aged 65 years and older [1], [2] and constitutes the most common form of dementia among older adults. While several major genes contributing to risk of Alzheimer Disease have been identified (*APP* [3], *PS1* [4], *PS2* [5]–[7]), all but one (*APOE* [8]–[10]) contributed predominantly to early-onset forms of AD that cluster within families; other than *APOE*, few consistent association signals have been observed for late-onset AD (LOAD). Recent estimates of the heritability of LOAD fall between 60% and 80% [11]. However, while *APOE* ε4-alleles elevate AD risk, only 50% of AD cases carry an *APOE* ε4 allele, suggesting genetic factors elsewhere in the genome contribute to AD risk [12].

At present, eleven studies have tested association with LOAD on genome-wide panels of single nucleotide polymorphisms (SNPs). Most [13]–[22], but not all [23], of these studies indirectly observed associations with APOE on chromosome 19q with strong experiment-wide statistical significance. However, only a few of the studies observed associations at other loci exceeded experiment-wide statistical significance thresholds. A follow-up study [15] to Coon et al. [14] stratifying cases and controls by *APOE* genotype detected strong associations with *GAB2* (MIM:606203) SNPs, and in follow-up work observed altered *GAB2* transcript levels in vulnerable neurons, and an effect of *GAB2* levels on tau phosphorylation; replication studies observed mixed results. In a family-based study of LOAD, Bertram et al. [17] observed four SNP associations exceeding adjusted experiment-wide thresholds for statistical significance, including one for the chromosome. Our group reported a SNP association with experiment-wide statistical significance on chromosome 12q13 [18]. A GWAS originating from the Mayo Clinic [19] identified a novel signal on the X chromosome in the gene *PCDH11X* (MIM: 300246), encoding a protocadherin, a cell-cell adhesion molecule expressed in the brain. Generally, these earlier reports have not been consistently replicated in other studies, possibly due to sample sizes that are substantially smaller than those of GWAS studies that have successfully identified genes for other complex disorders [24], [25]. Two large collaborative GWAS of LOAD examined many thousands of cases and controls [20], [21] and both identified novel association signals in the gene *CLU* (aka *APOJ*, MIM: 185430; Apolipoprotein J or Clusterin), as well as signals in *CR1* (MIM: 120620, Complement Component Receptor 1) and in *PICALM* (MIM: 603025, Phosphatidylinositol-Binding Clathrin Assembly Protein), reporting some of the most consistent results for LOAD to date.

Even with the increased sample sizes and improved statistical power to detect loci with moderate effect sizes, it remains unlikely that these studies, incorporating cases and controls from multiple samples with varying case/control inclusion criteria, have identified all loci with modest effect sizes in LOAD. We analyzed genome-wide association in a discovery dataset of 931 cases and 1,104 controls and performed replication analysis on the strongest associations (*P*<10^−5^) using genotype data from four existing studies totaling 1,338 cases and 2,003 controls.

## Results

### Dataset Characteristics

Table 1 depicts the demographic characteristics of the case and control samples examined in initial association analyses. We examined 931 LOAD cases, average age 74.4 years at onset (standard deviation: ±8.1 years), and 1,104 cognitive controls, average age 73.8 years at exam (±7.8 years) (Table 1). Cases were 64.5% female, while controls were 61.9% female.

**Table 1:** Demographic characteristics of participants in the study sample (mean ± SD or number (percent)).

	All	Cases	Controls
**Number of subjects**	2,036	931	1,104
Females (%)	1,284 (63.0%)	601 (64.5%)	683 (61.9%)
Age-at-onset [cases] (yr)/Age-at-exam [controls] (yr)	–	74.4±8.1	73.8±7.8
**APOE ε4 carrier status**			
–/– carriers (0 copies)	1223 (60.1%)	399 (42.8%)	824 (74.6%)
ε4/– carriers (1 copy)	629 (30.9%)	398 (42.7%)	231 (20.9%)
ε4/ε4 carriers (2 copies)	144 (7.1%)	127 (13.6%)	17 (1.5%)
carrier status missing	40 (2.0%)	8 (0.9%)	32 (2.9%)

*Percentage of successfully genotyped single nucleotide polymorphisms (SNPs) among those attempted.

### GWAS Results

**Figure 1:**
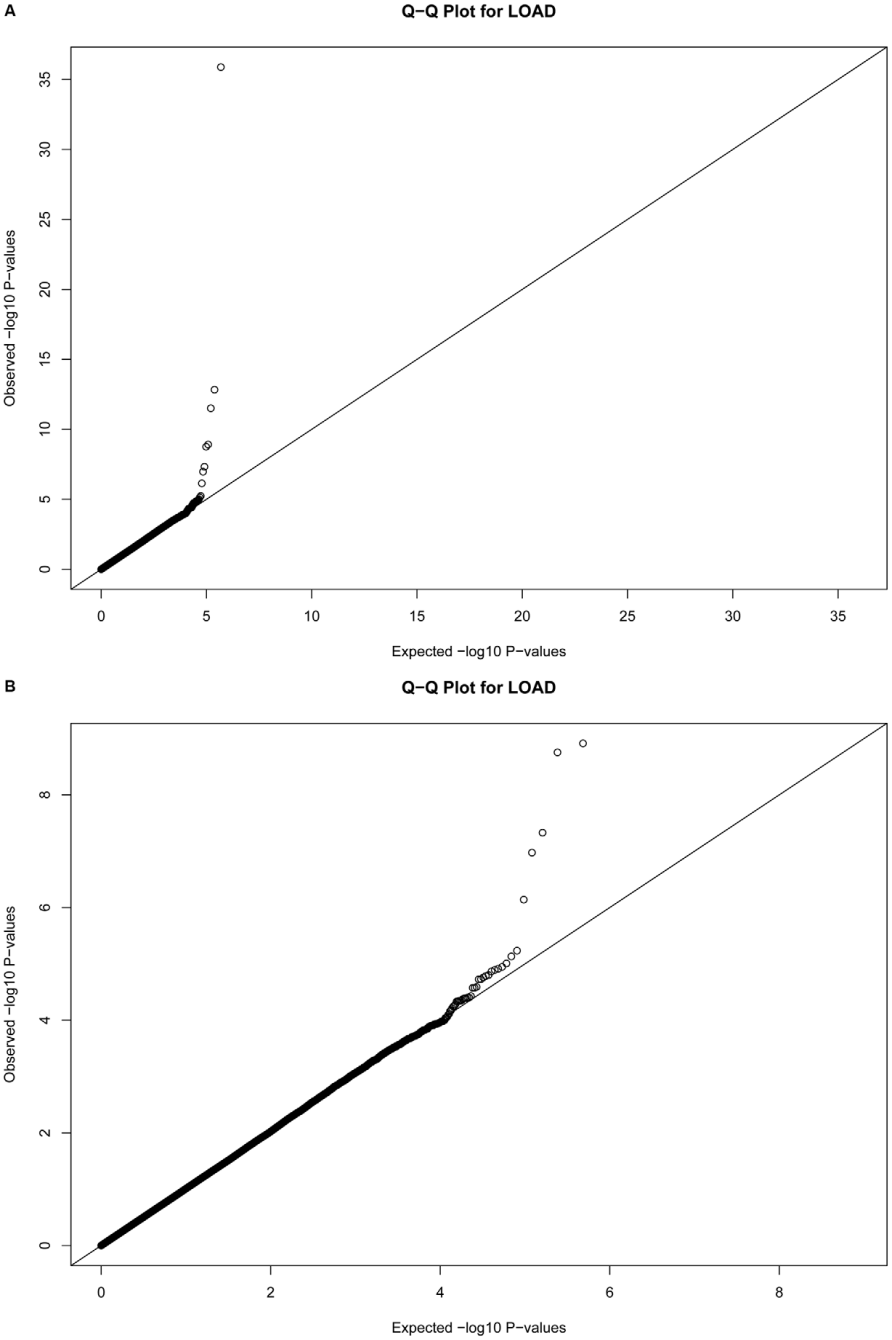
Quantile-Quantile plots for 483,399 single SNP tests of association. Plots depict expected versus observed −*log*10 P-values for 483,399 single SNP tests of association (in 931 LOAD cases and 1,104 cognitive controls, with adjustment for principal components as covariates for population substructure). Plot **A** includes the most-strongly associated SNPs within the APOE locus, whereas plot **B** excludes the three most-strongly associated SNPs for clarity.

11 SNPs had association p-values (*P*)<10^−5^ after adjustment for population substructure (Table 2; Q-Q plot for all association results in Figure 1; *P*<10^−4^ in Tables S1, S2; all association results in Figure S1). Although the SNPs defining the *APOE* ε2, ε3, and ε4 alleles, rs429358 and rs7412, were not included on our genotyping platforms, we independently genotyped these SNPs and tested the association of *APOE* ε4 with LOAD risk (OR (95% CI): 4.18 (3.51, 4.97); 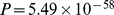). SNPs adjacent to the *APOE* haplotype on chromosome 19 otherwise demonstrated the highest associations observed, with the peak association being rs2075650 with 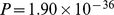, confirming the expected effect of *APOE* on LOAD risk in this sample. The most significant non-*APOE* SNP in our previous GWAS [18] (Table S3) was rs11610206 on 12q13 (45.92 Mb) with 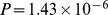; in this study, this SNP was still strongly assoFciated with LOAD (OR (95% CI): 0.67 (0.54, 0.85); 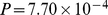), but not with experiment-wide statistical significance.

**Table 2:** The strongest associations (*P*<10^−5^) from a GWAS of late-onset Alzheimer disease.

						Discovery GWAS		Replication GWAS		Combined GWAS	
SNP	Chr	Location	Gene***	Function***	Minor Allele (Freq.)	OR* (95% CI**)	P	OR* (95% CI**)	P	OR* (95% CI**)	P
rs2075650	19	50087459	*TOMM40*	intron	G (0.2)	2.96 (2.50, 3.50)	1.30×10∧−36	5.72 (3.63, 9.02)	6.24×10∧−14	2.94 (2.48, 3.47)	4.87×10∧−36
rs405509	19	50100676	*APOE*		C (0.48)	0.62 (0.55, 0.70)	1.47×10∧−13	0.65 (0.49, 0.87)	0.00342	0.61 (0.54, 0.70)	8.13×10∧−14
rs8106922	19	50093506	*TOMM40*	intron	G (0.36)	0.62 (0.54, 0.71)	3.10×10∧−12	0.79 (0.6, 1.05)	0.108	0.62 (0.54, 0.71)	2.94×10∧−12
rs157580	19	50087106	*TOMM40*	intron	G (0.35)	0.66 (0.57, 0.75)	1.22×10∧−9	0.49 (0.36, 0.68)	0.0000153	0.63 (0.55, 0.71)	7.78×10∧−13
rs439401	19	50106291	*LOC100129500*	intron	A (0.34)	0.66 (0.57, 0.75)	1.76×10∧−9	0.33 (0.22, 0.51)	4.23×10∧−7	0.63 (0.55, 0.72)	3.80×10∧−12
rs11754661	6	151248771	*MTHFD1L*	intron	A (0.07)	2.03 (1.58, 2.62)	4.70×10∧−8	2.34 (1.37, 3.98)	0.00187	2.10 (1.67, 2.64)	1.90×10∧−10
rs6859	19	50073874	*PVRL2*	intron	A (0.46)	1.41 (1.24, 1.60)	1.06×10∧−7	1.70 (1.35, 2.13)	6.13×10∧−6	1.41 (1.24, 1.60)	9.60×10∧−8
rs10402271	19	50021054			C (0.36)	1.39 (1.22, 1.59)	7.26×10∧−7	1.23 (1.1, 1.38)	0.000277	1.26 (1.16, 1.38)	2.14×10∧−7
rs6509916	19	60254214	*RDH13*	intron	G (0.46)	1.34 (1.18, 1.52)	5.83×10∧−6	0.87 (0.78, 0.98)	0.0223	1.10 (1.01, 1.20)	0.0334
rs509512	11	105350133	*GRIA4*	intron	C (0.43)	0.75 (0.66, 0.85)	7.37×10∧−6	1.04 (0.94, 1.16)	0.439	0.94 (0.86, 1.02)	0.133
rs679670	6	138179244			G (0.37)	0.74 (0.65, 0.85)	9.83×10∧−6	1.11 (0.93, 1.34)	0.25	0.87 (0.78, 0.97)	0.016

*OR = Odds Ratio.

**CI = Confidence Interval.

***Gene Annotation using SNPper database [62].

Single nucleotide polymorphisms (SNPs) demonstrating association with late-onset Alzheimer Disease at *P*<10^−5^ in association tests adjusting for covariates from principal components capturing population substructure, evaluated in the Discovery genome-wide association study (GWAS) dataset of 931 independent cases and 1,104 independent cognitively normal controls, in the Replication GWAS dataset of 1,242 independent cases and 1,737 independent controls, and in the Combined GWAS dataset of 2,174 cases and 2,181 controls.

The SNP rs11754661, located at 151.2Mb of chromosome 6q25.1 in the gene *MTHFD1L*, was significantly associated with LOAD (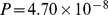; Bonferroni-corrected *P* = 0.022). To ensure that this association was not spurious due to differences between subsets of genotyped samples, we performed several post-hoc quality control analyses. We examined clustering plots from genotype calling by platform to determine if misclassification could have affected the associations observed for the top 11 SNPs with *P*<10^−5^, and observed discrete clustering by genotype for all 11 SNPs. We found no evidence of a difference in genotype frequencies among controls across subsets by genotyping platform (Fisher's exact test *P* = 0.71) or by study center (Fisher's exact test *P* = 0.95, Table S4). We also examined differences in dataset characteristics including variation in age, sex, and *APOE* ε4 genotype distributions, and found limited differences between subsets by study center, autopsy- or clinical-confirmation of case or control status, and by genotyping platform (Table S5). Subsequently, we examined the first hundred principal components generated from EIGENSTRAT to determine if any of the principal components were associated with both differences in genotyping platform subset and disease status at *P*<0.05 as markers of potential systematic bias. While two principal components other than those used to adjust for population substructure showed association with both genotyping platform subset and LOAD, additional adjustment for these principal components did not change the strength of association between rs11754661 and LOAD (data not shown). Models further adjusting for age, sex, and *APOE* ε4 carrier status (+/−) (Table S6) only marginally diminished the effect size and statistical significance of the association of rs11754661 with LOAD (adjustment for age and sex, OR (95% CI): 2.03 (1.56, 2.64), 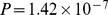; adjustment for age, sex and *APOE* ε4 (+/−), OR (95% CI): 2.01 (1.51, 2.67), 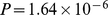).

**Figure 2:**
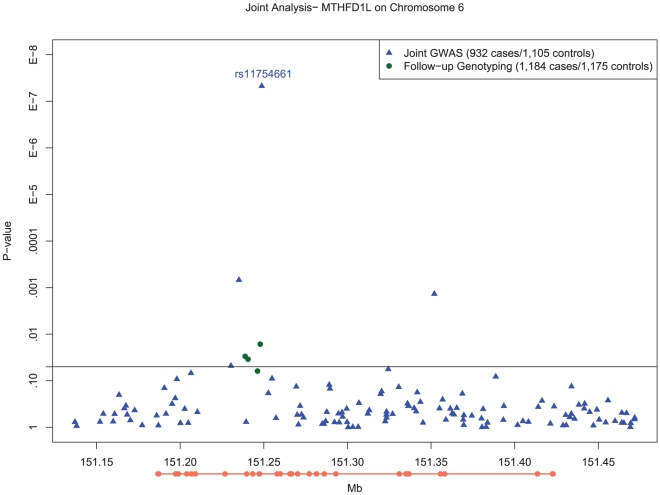
Manhattan plot of SNP associations in *MTHFD1L*, on chromosome 6 between 151.2 Mb and 151.3 Mb. Plot of −*log*10 P-values for single SNP tests of association with LOAD with adjustment for population substructure for the chromosome 6 region from 151.2Mb to 151.3Mb in *MTHFD1L*. Blue circles depict results for SNPs examined as part of genomewide association testing, whereas the green circle depicts the genome wide significant association of rs11754661 and the green triangles show the association results of the additional six SNPs proximal to rs11754661 genotyped on the Taqman platform. The orange line below the x-axis depicts the exons (thick line) and introns (thin line) of the *MTHFD1L*, oriented 5′ to 3′ from left to right.

Furthermore, we examined the associations in 4 SNPs in linkage disequilibrium (LD) (Figures S2) of *D'*>0.8 with rs11754661, which demonstrated variable patterns of association with LOAD (Figure 2; rs2839947, *P* = 0.0479; rs11757561, *P* = 0.000684; rs2073066, *P* = 0.768; rs13201018, *P* = 0.185). It should be noted that due to the low minor allele frequency (MAF) of rs11754661 (MAF = 0.07), only one of these SNPs, rs11757561 (MAF = 0.20), had an *r^2^*>0.10 (*r^2^* = 0.23). This SNP had a similar direction of association as rs11754461 (OR (95% CI): 1.31 (1.12, 1.53)).

Based on the pattern of LD in the vicinity of rs11754661, we examined several haplotypes of *MTHFD1L* which included this SNP (described in Text S1) to identify potential markers for untyped variants associated with LOAD. Two haplotypes (the first comprising rs2073066-rs11754661-rs13201018, the second comprising rs2839947-rs11757561-rs2073066-rs11754661-rs13201018) both containing the risk-increasing A allele of rs11754661 had highly statistically significant associations similar to the genotypic association of rs11754661 (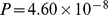 and 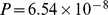, respectively) (Table S7). Both haplotypes had similar frequencies (MHF) to the A allele of rs11754661 (MHF = 0.0696 and MHF = 0.0629, respectively).

In order to ensure that the association we observed at rs11754661 was not merely due to genotyping error, we genotyped four additional SNPs in *MTHFD1L* proximal to and in high LD (*r^2^*>0.8) with rs11754661. All SNPs but one (rs7765521, *P* = 0.055) demonstrated associations with nominal statistical significance (rs803424, *P* = 0.016; rs2073067, *P* = 0.030; and rs2072064, *P* = 0.035). Figure 2 shows the *−log*10-transformed P-values for single SNP tests of association in the *MTHFD1L* and 50kb flanking region (151.2Mb–151.3Mb) surrounding the chromosome 6 association signal at rs11754661, among both SNPs genotyped in the initial GWAS and those genotyped subsequently.

Association analyses of pooled datasets combining data on 1,242 cases and 1,737 controls confirmed experiment-wide statistically significant associations for SNPs in/near *APOE* (replication from 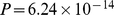 to *P* = 0.00187) for all but one SNP (rs8106922; discovery 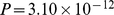, replication *P* = 0.108) (Table 2), however the direction of association in the replication was consistent across each of these SNPs. The association of the *MTHFD1L* SNP rs11754661 in the replication was both statistically significant (*P* = 0.00187) and showed similar strength and direction (discovery OR (95%CI): 2.03 (1.58, 2.62); replication OR (95%CI): 2.34 (1.37, 3.98)).

### Association in Combined Discovery and Replication Datasets

In combined analyses, associations in and around the *APOE* locus were unequivocally strengthened, with the p-values observed ranging from 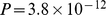 to 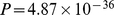. Variation at rs11754461 was strongly associated (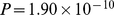) with an elevated risk of LOAD with OR = 2.10 (95% CI: 1.67, 2.64). Several adjacent SNPs also demonstrated nominal associations with similar direction of effect, including rs11757561 (*P* = 0.000846) with OR = 1.31 (95% CI: 1.12, 1.53) and rs12195069 (P = 0.0432) with OR = 1.25 (95% CI: 1.01, 1.55).

Two SNPs with only modest statistical significance of association in the discovery GWAS demonstrated highly statistically significant association in analyses combining both discovery and replication datasets (Tables S1 and S2). SNPs rs4676049 and rs17034806, located at 109Mb on chromosome 2q13, had associations of OR = 1.62 (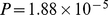) and OR = 1.61 (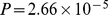) respectively in the discovery dataset. However, combining discovery and replication datasets, the SNP associations gained modest strength in effect size (OR = 1.76 for rs4676049 and OR = 1.75 for rs17034806), but the associations now exceeded the threshold for experiment-wide statistical significance, with 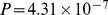 for rs4676049 and 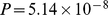 for rs17034806.

## Discussion

Although associations with experiment-wide statistical significance have not been observed for *MTHFD1L* in previous GWAS of LOAD, biological evidence suggests a role for this gene in dementia and AD pathology. *MTHFD1L*, which encodes the methylenetetrahydrofolate dehydrogenase (NADP+ dependent) 1-like protein, is involved in tetrahydrofolate (THF) synthesis, catalyzing the reversible synthesis of 10-formyl-THF to formate and THF, an important step in homocysteine conversion to methionine [26]. Elevated plasma homocysteine levels have been implicated in AD [27], [28] and other neurodegenerative disease including Parkinson's [29], and have been recognized as a risk factor for pre-eclampsia [30], diabetic complications [31], and heart disease [32]. Interestingly, a recent GWAS of coronary artery disease (CAD) identified *MTHFD1L* as a CAD risk factor in both British and German populations studied [33]. Several potential mechanisms may explain this connection: hyperhomocysteinaemia may influence AD dementia by causing vascular alterations [34]; it may cause cholinergic deficit due to toxicity to cortical neurons [35]; several lines of evidence suggest that elevated homocysteine contributes to AD risk through increased oxidative stress [36]–[38]. On-going biological investigations are continuing to elucidate the pathways connecting elevated homocysteine with AD.

Mthfd1l protein has been reported to be decreased in the hippocampus in a mouse model of AD using a proteomic approach [39]. Homocysteic acid, derived from homocysteine and methionine, is elevated in these mice and treatment with antibodies to homocysteic acid reduced amyloid burden and inhibited cognitive decline in these animals [40]. B6-deficient diets lead to further increases in homocysteic acid in these mice.

That we observed an experiment-wide statistically significant association in *MTHFD1L* in addition to the associations of a number of *APOE* SNPs with LOAD risk is consistent with results from previous work. *MTHFD1L* is located on chromosome 6q25.1, near linkage signals observed in two prior genome-wide linkage studies of LOAD [41], [42]. The previous GWAS performed by our group [18], from which nearly 1,000 individuals in the current study were drawn, observed a strong, but not experiment-wide, statistically significant association between the same *MTHFD1L* SNP and LOAD at *P* = 2.01×10^−5^. Experiment-wide statistical significance for this association was observed with the addition of another 1,047 individuals in this study.

We did not observe associations with LOAD with experiment-wide statistical significance in any of the peak non-*APOE* signals identified in previous GWAS studies, including the *APOJ*/*CLU* SNP rs11136000 that was identified in both Harold et al. and Lambert et al. studies (analysis of this dataset reported elsewhere (Jun et al., in preparation)). Given the observed OR = 0.86 of rs11136000 for LOAD, in our sample of 931 cases and 1,104 controls, we had <1% power to detect the observed effect at the Bonferroni-corrected threshold for experiment-wide statistical significance, α = 1.03×10^−7^, suggesting that most significant associations of small or modest effect size would be missed in this study. The association of variation in *PCDH11X* and *GAB2* was not observed in this dataset; the findings of these analyses are reported elsewhere [43]. In addition, we observed a strong association of the chromosome 12 SNP rs11610206 with LOAD, but not with genomewide statistical significance as observed in our previous GWAS [18], suggesting that the findings of the Beecham et al. study, as with previous LOAD GWAS, may be subject to the “winner's curse” [44].

Despite a wealth of evidence for the role of chromosome 2 loci in Alzheimer's Disease, the chromosome 2q13 SNPs identified with experiment-wide statistically significant associations in the combined analyses, rs4676049 and rs17034806, do not fall in the vicinity of chromosome 2 regions of interest [22], .

Based on the patterns of studies emerging in other complex diseases, GWAS studies with sample sizes greater than the combined Lambert et al. and Harold et al. datasets may be necessary to validate associations observed in smaller GWAS studies and to identify susceptibility variants with more modest effects. This approach has been taken in type 2 diabetes, where a meta-analysis of 54,000 subjects confirmed multiple susceptibility loci [43]. Other approaches to identify new susceptibility variants are exploring the Common Disease-Rare Variants (CDRV) hypothesis, which aim to identify novel susceptibility loci for disease by assessing the aggregate effects of multiple rare variants in single genes on disease risk [46].

In this genome-wide association study of LOAD, we identified a novel association with experiment-wide statistical significance in a gene with a potential biological role, *MTHFD1L*. We replicated this association in additional publicly-available genomewide association datasets, and observed statistically significant association with a similar effect size and direction at this SNP. In summary, *MTHFD1L* is an excellent candidate for LOAD on account of its involvement in folate-pathway abnormalities linked with homocysteine, a significant biological risk factor for AD.

## Methods

### Ethics Statement

After complete description of the study to the subjects, written informed consent was obtained from all participants, in agreement with protocols approved by the institutional review board at each contributing center.

### Ascertainment

Discovery dataset cases and controls were clinically ascertained through the Collaborative Alzheimer's Project (CAP) comprising the University of Miami John P. Hussman Institute for Human Genomics (HIHG) and the Vanderbilt University Center for Human Genetics Research (CHGR), and autopsy-verified cases and controls were collected through the Mount Sinai Brain Bank (MSBB) at the Mount Sinai School of Medicine (see [47]). Additional controls were also identified in the National Cell Repository for Alzheimer's Disease (NCRAD). 266 cases and 643 controls genotyped in the discovery dataset from the NCRAD, HIHG, and CHGR [48] and NCRAD were independent from previously published data sets including those from our group's previously published GWAS [18]. All CAP-ascertained cases and controls were recruited and evaluated using standardized criteria and protocols, and case adjudication in the CAP was performed jointly by a Clinical Advisory Board (CAB) composed of both HIHG and CHGR members, with controls evaluated jointly as well.

All cases and controls from the HIHG, CHGR, and NCRAD met selection criteria described in the Beecham et al. study [18]. Briefly, the study was described and written informed consists were obtained from all participants, in accordance with institutional review board protocols at each study center. Each individual classified as a LOAD case met the NINCDS-ADRDA criteria for probable or definite AD and had an age at onset greater than 60 years of age [49], as determined from specific questions within the clinical history answered by a reliable family informant or from documented significant cognitive impairment in the medical record. Vascular dementia was diagnosed according to contemporary standards [50] by the CAB, and individuals with confirmed vascular dementia or phenotypic uncertainty were excluded from analyses. Cognitive controls were individuals who showed signs of dementia in clinical history or upon interview, and were drawn from spouses, friends, and other biologically unrelated individuals of cases, were frequency-matched by age and gender to the cases, and were located in the same clinical catchment areas. All cognitive controls were examined, and none showed signs of dementia in clinical history or upon interview. Also, each cognitive control had a documented Mini-Mental State Exam (MMSE) score ≥27 or a Modified Mini-Mental State (3MS) Exam score ≥87. Clinical history and interview data for NCRAD controls, including MMSE scores, were made available and collected along with whole blood for DNA extraction for inclusion in our study.

306 cases and 81 controls identified in the MSBB were recently deceased patients at the Mount Sinai Medical Center in New York, NY, and had affection status verified through clinical review and brain autopsy. Neither the cases nor controls examined have been used in previously published studies. Covariates including age at death and sex were abstracted from reviews of medical charts performed by members of the MSBB.

In total, 572 new cases and 724 new controls were genotyped in this study, and after quality controls measures, combined with data on 492 cases and 496 controls from the previous GWAS [18] for analysis. We also had available for replication from the HIHG, a dataset of 246 cases and 69 cognitively normal controls from a previously described dataset [51].

### Genotyping

We extracted DNA for individuals ascertained by the HIHG, CHGR, MSBB, and NCRAD from whole blood by using Puregene chemistry (QIAGEN, Germantown, MD, USA). We performed genotyping using the Illumina Beadstation and the Illumina Infinium Human 1M beadchip on 530 cases and 393 controls following the recommended protocol, only with a more stringent GenCall score threshold of 0.25. Genotyping on 248 controls from the PD GWAS dataset [48] was performed using the Illumina Infinium Human 610-Quad beadchip. Genotyping efficiency was greater than 99%, and quality assurance was achieved by the inclusion of one CEPH control per 96-well plate that was genotyped multiple times. Technicians were blinded to affection status and quality-control samples. We used Taqman Genotyping Assays for SNPs +3937/rs429358 and +4075/rs7412 and performed allelic discrimination/genotype calling on the ABI 7900 Taqman system, the results of which were used to determine *APOE* ε2/ε3/ε4 genotypes.

After excluding samples which failed quality control (described in the next section) with low genotyping call rates, genotype data was available on 870,954 SNPs (after quality control) using the Illumina 1M BeadChip on 440 cases and 437 controls, while genotype data on 490,960 SNPs (after quality control) from the Illumina 610Quad BeadChip was available on 172 controls. Combining these data with the 522,366 SNPs on 492 cases and 496 controls in our previous GWAS [18], a set of 483,399 SNPs common to all platforms was generated that passed quality control for each subset individually and in a pooled dataset. The Bonferroni-corrected threshold for experiment-wide statistical significance was thus set at Bonferroni-corrected 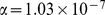.

### Sample Quality Control

After genotyping, multiple quality controls were performed including assessment of sample efficiency, which is the proportion of valid genotype calls to attempted calls within a sample. Samples with efficiency less than 0.98 were dropped from the analysis. Reported gender and genetic gender were examined with the use of X-linked SNPs; 32 inconsistent samples were dropped from the analysis. Relatedness between samples was tested via the program Graphical Representation of Relatedness (GRR) [52], and 3 related samples were dropped from the analysis.

To determine if population substructure exists in the case-control sample, a set of 10,000 SNPs with MAF>0.25, selected for minimal between-SNP linkage disequilibrium (*r^2^*<0.20), and spread evenly across the autosomal chromosomes were analyzed using the program STRUCTURE [53], [54] (burn in: 5,000, iterations: 25,000) assuming different number of assumed subpopulations (K). The −log likelihood for K was maximized at K = 3, suggesting population substructure. Further analysis was performed in EIGENSTRAT [55], where principal components analysis on the sample of 10,000 SNPs was used to generate principal component loadings for samples and remove outliers by using the top ten principal components over 5 iterations with a threshold of six standard deviations. The top three principal component loadings were used as covariates to account for population structure in the association analysis.

Removing genotyped individuals with low genotype call rates, incorrect reported gender, high relatedness with other samples, and extreme outliers in substructure analyses, 440 cases and 608 controls remained for inclusion in analysis, and were combined with 492 cases and 496 controls from the previous GWAS.

### SNP Quality Control

Quality control was performed to remove any low quality SNPs. Genotype clusters were redefined using signal intensities of samples with efficiency greater than 0.98, and genotypes were recalled on the basis of these new clusters per the manufacturer's recommendation. Efficiency of individual SNPs was estimated as the proportion of samples with genotype calls for a given SNP, and SNPs with efficiency less than 0.95 were dropped from analysis. Due to concerns of low statistical power to detect association, SNPs with MAF<0.005 were dropped from analysis. Hardy-Weinberg Disequilibrium (HWD) statistics were calculated among controls with the Fisher's exact test in the PLINK software package [56]; SNPs with *P*<10^−6^ for HWD were dropped from analysis. In addition, due to concerns with the spurious association originating from the use of different genotyping platforms on samples in the previous and current GWAS studies, distributions of genotype frequencies at each SNP in each study were examined among controls using a Fisher's exact test, and SNPs with highly-differing genotype distributions across genotyping subsets (*P*<0.001) were dropped prior to analysis. After these quality control measures, 483,399 SNPs remained for association analysis.

### Association Analysis

Association analysis was performed using logistic regression to test association of genotypes with LOAD under an additive model. Logistic regression was used to permit covariate adjustment for loadings taken from the first three principal components identified in EIGENSTRAT to account for population substructure. Here we report results from logistic regression models adjusting only for population substructure with principal components. Further regression modeling was also performed on SNPs with initial associations of *P*<10^−5^, extending models to adjust for *APOE* genotype (designated as the number of ε4 alleles), age-at-onset in cases and age-at-exam in controls, and gender as covariates (Table S6). All analyses were performed using the PLINK software package [56].

Quantile-quantile plots of the associations were made (Figure 1), and suggest the absence of systematic bias in the tests of association.

### Imputation and Replication Analysis

To provide independent replication of the associations observed in the discovery dataset, genome-wide genotyping data were combined from four additional datasets (one unpublished and three publicly-available datasets) and missing genotype data imputed using IMPUTE v1.0 [57] (Table S8). SNPs with differing genotypic distributions between datasets were excluded from imputation using the Fisher's exact test approached described earlier [58]. Both primary and replication datasets were imputed to a HapMap reference of over 2.5 million SNPs. Individual genotypes with probability less than 0.90 were not included, and SNPs missing >10% of genotypes within either data set were dropped. In addition to using the combined Hapmap Phase III CEPH Utah pedigree (CEU) and Tuscan (TSI) haplotype reference panels for imputation, for imputation within each study, we used genotype data on controls from other datasets to improve imputation accuracy, and Affymetrix 5.0 genotype data on 105 individuals genotyped in an independent Ashkenazi Jewish genotyping panel [59].

We analyzed existing pooled and imputed datasets of unrelated individuals from several studies: 147 cases and 182 controls from the Alzheimer's Disease Neuroimaging Initiative (ADNI) [60], 86 cases and 1,200 controls (all unrelated) from the Framingham Study SHARe dataset [61], and 859 cases and 552 controls from the Reiman et al. [15] LOAD GWAS dataset, and a set of 246 LOAD cases and 69 cognitively normal controls previously described [51] and genotyped on the Affymetrix 6.0 genotyping platform on which results have not been previously published.

## Supporting Information

Figure S1 Plots of −*log*10 *P*-values for 483,399 single SNP tests of association (in 931 LOAD cases and 1,104 cognitive controls, with adjustment for principal components as covariates for population substructure). Plot A includes association results from all SNPs within the APOE locus, whereas plot B excludes the three most strongly associated SNPs for clarity.
(3.45 MB TIF)

Figure S2 LD (Plot A: *D'*, Plot B: *r^2^*) between 130 SNPs genotyped in 931 cases and 1,104 controls in and around the gene *MTHFD1L* (±50 kilobasepairs). The SNP with the most significant association, rs11754661, is highlighted with a blue arrow in the diagram below.
(2.01 MB TIF)

Table S1 Single nucleotide polymorphisms (SNPs) demonstrating association with late-onset Alzheimer Disease at *P*<10^−4^ in association tests adjusting for covariates from principal components capturing population substructure, evaluated in the Discovery genome-wide association study (GWAS) dataset of 931 independent cases and 1,104 independent cognitively normal controls, in the Replication GWAS dataset of 1,242 independent cases and 1,737 independent controls, and in the Combined GWAS dataset of 2,174 cases and 2,181 controls.
(0.11 MB DOC)

Table S2 Genotyped and imputed single nucleotide polymorphisms (SNPs) demonstrating association with late-onset Alzheimer Disease at *P*<10^−4^ in association tests adjusting for covariates from principal components capturing population substructure, evaluated in the Discovery genome-wide association study (GWAS) dataset of 931 independent cases and 1,104 independent cognitively normal controls, in the Replication GWAS dataset of 1,242 independent cases and 1,737 independent controls, and in the Combined GWAS dataset of 2,174 cases and 2,181 controls.
(0.30 MB DOC)

Table S3 Follow-up of the strongest associations reported in the Beecham, et al.(2009) [18] GWAS of late-onset Alzheimer Disease. 32 single nucleotide polymorphisms (SNPs) demonstrating the strongest association with late-onset Alzheimer Disease at *P*<10^−5^ in the Beecham et al. (2009) [18] GWAS of late-onset Alzheimer's Disease, tested here for association with adjustment for covariates from principal components capturing population substructure, evaluated in the Discovery genome-wide association study (GWAS) dataset of 931 independent cases and 1,104 independent cognitively normal controls.
(0.06 MB DOC)

Table S4 Genotype frequency distributions and differences in three subsets of a GWAS dataset for SNPs with strong associations with late-onset Alzheimer Disease. Genotype counts, *P*-values for Hardy-Weinberg Equilibrium (HWE), and *P*-values for differences in genotypic distribution from a Fisher's Exact Test (FET) comparing controls in SNPs with strong associations with late-onset Alzheimer Disease in three subsets of a GWAS dataset: cognitively normal controls from the previously published Beecham et al (2009) study [18] (“Beecham et al. controls”), cognitively normal controls recruited after the Beecham et al. (2009) study (“New AD Controls”), and cogntively normal controls consented for multiple genetic studies whose recruitment was funded through the Udall Parkinson's Disease Collaboration (“Udall Controls”).
(0.05 MB DOC)

Table S5 Demographic characteristics of participants, subsetted by study center, autopsy or clinical confirmation of case or control status, and by genotyping platform (mean ± SD or number (percent)).
(0.07 MB DOC)

Table S6 Changes in effect size and p-value with additional covariate adjustment for age, sex, and presence/absence of the APOE ε4 allele for SNP associations demonstrating *P*<10^−5^ in preliminary analyses of late-onset Alzheimer Disease. SNPs demonstrating association with late-onset Alzheimer Disease at *P*<10^−5^ as identified in Table 2, here showing results from logistic regression modeling with (1) no additional covariate adjustment, (2) additional covariate adjustment for age-at-onset (years, in cases only) and age-at-exam (years, in controls only) and sex, and (3) additional covariate adjustment for age-at-onset (years, in cases only) and age-at-exam (years, in controls only); sex; and presence presence/absence of the APOE ε4 allele. All models include, at minimum, covariate adjustment for principal components capturing population substructure.
(0.04 MB DOC)

Table S7 Associations with late-onset Alzheimer Disease of *MTHFD1L* haplotypes incorporating SNP rs11754661, with adjustment for covariates from principal components capturing population substructure, evaluated in the Discovery GWAS dataset of 931 independent cases and 1,104 independent cognitively normal controls.
(0.03 MB DOC)

Table S8 Genotyping or imputation of SNPs associated with LOAD at *P*<10^−4^. Index indicating whether single nucleotide polymorphisms (SNPs) demonstrating association with late-onset Alzheimer Disease at *P*<10^−4^ in association tests adjusting for population substructure in the Discovery dataset where genotyped or imputed in the Discovery dataset (931 independent cases and 1,104 independent cognitively normal controls) or any of the Replication datasets, including the from the Alzheimer's Disease Neuroimaging Initiative (ADNI) [60] (147 cases and 182 controls), the Framingham Study SHARe dataset (SHARe) [61] (86 cases and 1,200 controls (all unrelated)), the Reiman, et al., LOAD GWAS dataset (TGEN) [15] (859 cases and 552 controls), and an additional set of LOAD cases and controls independent of the Discovery dataset and not used in prior publications (ADRC) [51] (246 LOAD cases and 69 cognitively normal controls).
(0.24 MB DOC)

Text S1 Supplementary methods describing haplotype analyses.
(0.02 MB DOC)
