# Supplementary figures and images for: Dementia Revealed: Novel Chromosome 6 Locus for Late-Onset Alzheimer Disease Provides Genetic Evidence for Folate-Pathway Abnormalities
Source: PLoS Genet. 2010 Sep 23;6(9):e1001130. doi: 10.1371/journal.pgen.1001130 (PMC2944795; doi:10.1371/journal.pgen.1001130)

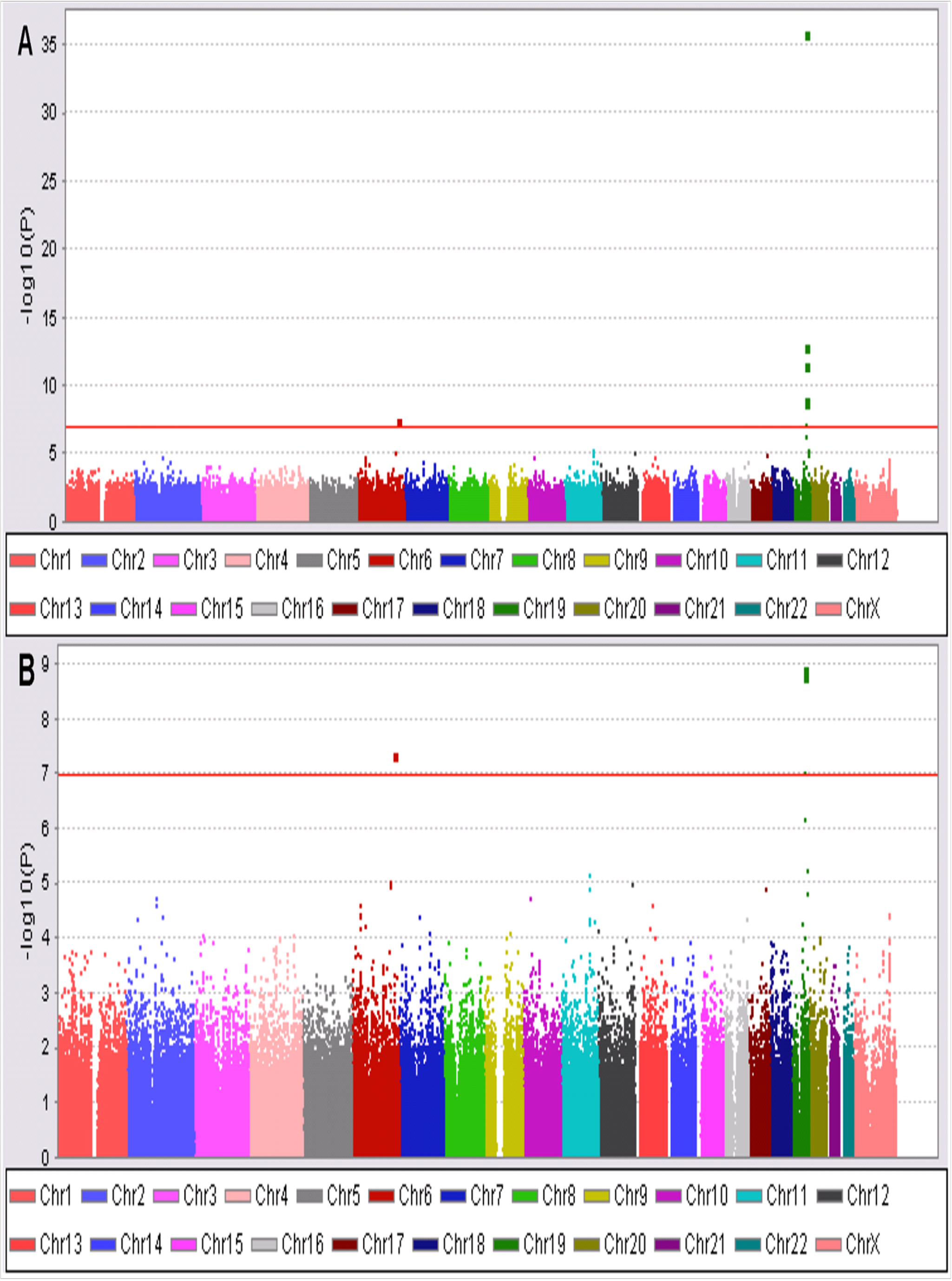

Supplement: Figure S1 — Plots of −log10 P-values for 483,399 single SNP tests of association (in 931 LOAD cases and 1,104 cognitive controls, with adjustment for principal components as covariates for population substructure). Plot A includes association results from all SNPs within the APOE locus, whereas plot B excludes the three most strongly associated SNPs for clarity. (3.45 MB TIF) [file pgen.1001130.s001.tif]

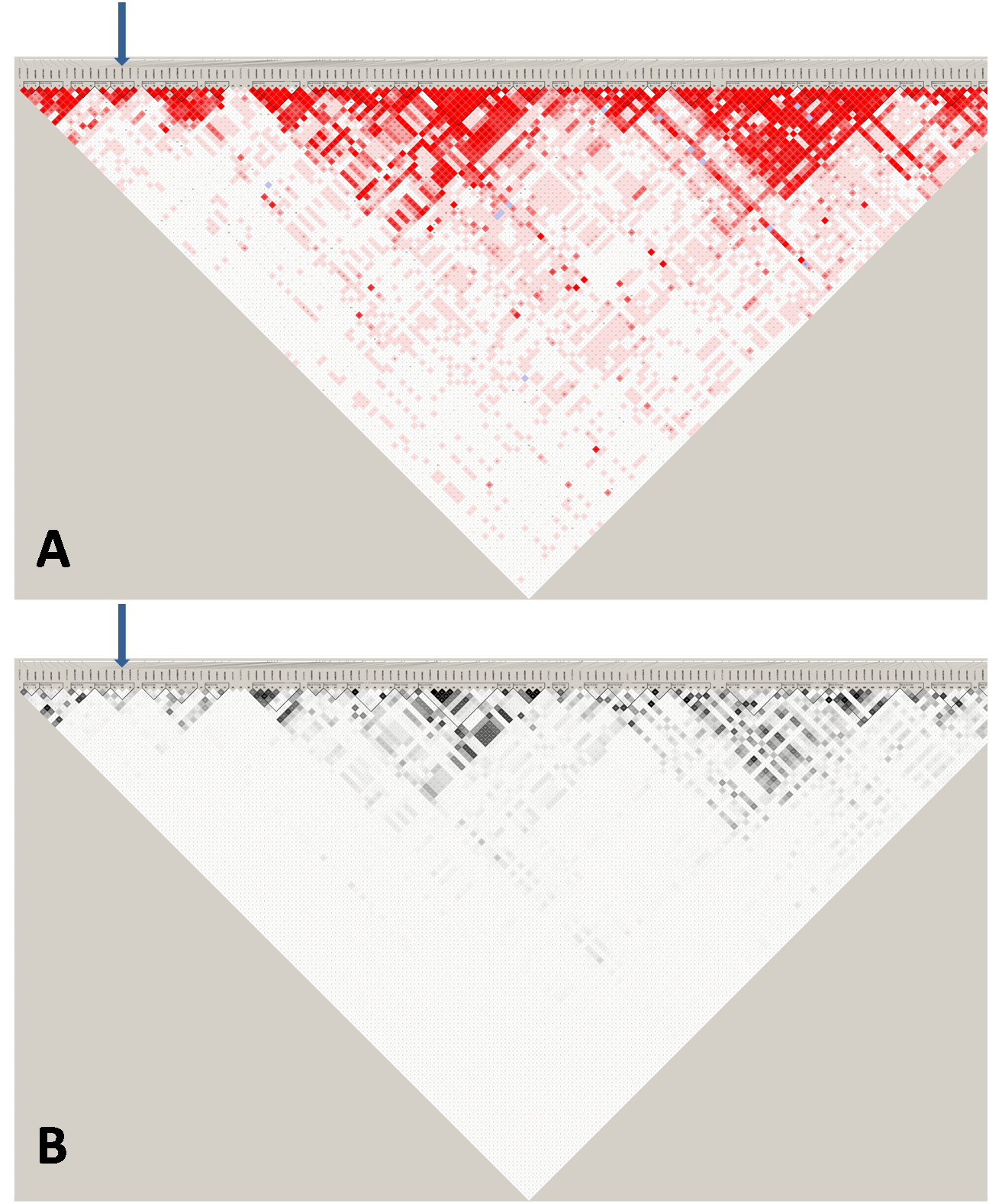

Supplement: Figure S2 — LD (Plot A: D', Plot B: r2) between 130 SNPs genotyped in 931 cases and 1,104 controls in and around the gene MTHFD1L (±50 kilobasepairs). The SNP with the most significant association, rs11754661, is highlighted with a blue arrow in the diagram below. (2.01 MB TIF) [file pgen.1001130.s002.tif]
