# Supplementary material for: Dementia Revealed: Novel Chromosome 6 Locus for Late-Onset Alzheimer Disease Provides Genetic Evidence for Folate-Pathway Abnormalities
Source: PLoS Genet. 2010 Sep 23;6(9):e1001130. doi: 10.1371/journal.pgen.1001130 (PMC2944795; doi:10.1371/journal.pgen.1001130)
Supplement: Table S1 — Single nucleotide polymorphisms (SNPs) demonstrating association with late-onset Alzheimer Disease at P<10−4 in association tests adjusting for covariates from principal components capturing population substructure, evaluated in the Discovery genome-wide association study (GWAS) dataset of 931 independent cases and 1,104 independent cognitively normal controls, in the Replication GWAS dataset of 1,242 independent cases and 1,737 independent controls, and in the Combined GWAS dataset of 2,174 cases and 2,181 controls. (0.11 MB DOC) [file pgen.1001130.s003.doc]

|  |  |  |  |  |  | **Discovery GWAS** | | **Replication GWAS** | | **Combined GWAS** | |
| --- | --- | --- | --- | --- | --- | --- | --- | --- | --- | --- | --- |
| **SNP** | **Chr** | **Location** | **Gene****** | **Function**  ******** | **Minor Allele (Freq.***)** | **OR* (95% CI**)** | **P** | **OR* (95% CI**)** | **P** | **OR* (95% CI**)** | **P** |
| rs2075650 | 19 | 50087459 | *TOMM40* | intron | G (0.2) | 2.96 (2.50, 3.50) | 1.30×10^-36 | 5.72 (3.63, 9.02) | 6.24×10^-14 | 2.94 (2.48, 3.47) | 4.87×10^-36 |
| rs405509 | 19 | 50100676 | *APOE* |  | C (0.48) | 0.62 (0.55, 0.70) | 1.47×10^-13 | 0.65 (0.49, 0.87) | 0.00342 | 0.61 (0.54, 0.70) | 8.13×10^-14 |
| rs8106922 | 19 | 50093506 | *TOMM40* | intron | G (0.36) | 0.62 (0.54, 0.71) | 3.10×10^-12 | 0.79 (0.60, 1.05) | 0.108 | 0.62 (0.54, 0.71) | 2.94×10^-12 |
| rs157580 | 19 | 50087106 | *TOMM40* | intron | G (0.35) | 0.66 (0.57, 0.75) | 1.22×10^-9 | 0.49 (0.36, 0.68) | 0.0000153 | 0.63 (0.55, 0.71) | 7.78×10^-13 |
| rs439401 | 19 | 50106291 | *LOC100129500* | intron | A (0.34) | 0.66 (0.57, 0.75) | 1.76×10^-9 | 0.33 (0.22, 0.51) | 4.23×10^-7 | 0.63 (0.55, 0.72) | 3.80×10^-12 |
| rs11754661 | 6 | 151248771 | *MTHFD1L* | intron | A (0.07) | 2.03 (1.58, 2.62) | 4.70×10^-8 | 2.34 (1.37, 3.98) | 0.00187 | 2.10 (1.67, 2.64) | 1.90×10^-10 |
| rs6859 | 19 | 50073874 | *PVRL2* | intron | A (0.46) | 1.41 (1.24, 1.60) | 1.06×10^-7 | 1.70 (1.35, 2.13) | 6.13×10^-6 | 1.41 (1.24, 1.60) | 9.60×10^-8 |
| rs10402271 | 19 | 50021054 |  |  | C (0.36) | 1.39 (1.22, 1.59) | 7.26×10^-7 | 1.23 (1.10, 1.38) | 0.000277 | 1.26 (1.16, 1.38) | 2.14×10^-7 |
| rs6509916 | 19 | 60254214 | *RDH13* | intron | G (0.46) | 1.34 (1.18, 1.52) | 5.83×10^-6 | 0.87 (0.78, 0.98) | 0.0223 | 1.10 (1.01, 1.20) | 0.0334 |
| rs509512 | 11 | 105350133 | *GRIA4* | intron | C (0.43) | 0.75 (0.66, 0.85) | 7.37×10^-6 | 1.04 (0.94, 1.16) | 0.439 | 0.94 (0.86, 1.02) | 0.133 |
| rs679670 | 6 | 138179244 |  |  | G (0.37) | 0.74 (0.65, 0.85) | 9.83×10^-6 | 1.11 (0.93, 1.34) | 0.25 | 0.87 (0.78, 0.97) | 0.016 |
| rs1244096 | 12 | 123481044 | *NCOR2* | intron | A (0.1) | 1.61 (1.30, 1.99) | 0.0000113 | 1.26 (0.95, 1.67) | 0.116 | 1.59 (1.29, 1.97) | 0.0000192 |
| rs799419 | 6 | 138180548 |  |  | G (0.37) | 0.74 (0.65, 0.85) | 0.0000122 | 1.12 (0.93, 1.35) | 0.223 | 0.87 (0.78, 0.98) | 0.0176 |
| rs669397 | 11 | 105351597 | *GRIA4* | intron | G (0.43) | 0.75 (0.66, 0.85) | 0.0000128 | 1.04 (0.93, 1.16) | 0.48 | 0.93 (0.86, 1.02) | 0.123 |
| rs8074294 | 17 | 61902137 | *PRKCA* | intron | G (0.39) | 0.75 (0.66, 0.85) | 0.0000136 | 1.08 (0.96, 1.21) | 0.193 | 0.94 (0.86, 1.03) | 0.198 |
| rs8113032 | 19 | 60245950 |  |  | A (0.41) | 1.32 (1.17, 1.50) | 0.0000158 | 0.87 (0.78, 0.97) | 0.0149 | 1.07 (0.98, 1.17) | 0.11 |
| rs7091819 | 10 | 26028836 |  |  | C (0.24) | 1.38 (1.19, 1.61) | 0.0000187 | 1.06 (0.94, 1.20) | 0.36 | 1.18 (1.07, 1.30) | 0.001 |
| rs4676049 | 2 | 109001689 |  |  | A (0.08) | 1.62 (1.30, 2.03) | 0.0000188 | 2.40 (1.57, 3.66) | 0.0000474 | 1.76 (1.44, 2.15) | 4.31×10^-8 |
| rs929156 | 6 | 30247678 | *TRIM15* | missense (S->N) | A (0.24) | 1.38 (1.19, 1.60) | 0.0000257 | 1.03 (0.90, 1.16) | 0.702 | 1.21 (1.09, 1.33) | 0.000238 |
| rs1360873 | 13 | 63489710 |  |  | A (0.18) | 0.70 (0.59, 0.83) | 0.0000265 | 0.98 (0.85, 1.14) | 0.831 | 0.86 (0.77, 0.96) | 0.00879 |
| rs17034806 | 2 | 109002337 |  |  | G (0.08) | 1.61 (1.29, 2.01) | 0.0000266 | 2.39 (1.56, 3.64) | 0.0000541 | 1.75 (1.43, 2.15) | 5.14×10^-8 |
| rs6903912 | 6 | 31099538 |  |  | A (0.23) | 0.72 (0.62, 0.84) | 0.0000373 | 0.95 (0.83, 1.09) | 0.483 | 0.84 (0.76, 0.94) | 0.00187 |
| rs5977248 | 23 | 129329168 | *SLC25A14* | intron | A (0.44) | 0.74 (0.65, 0.86) | 0.0000395 | -- | -- | 0.74 (0.64, 0.85) | 0.0000325 |
| rs2844779 | 6 | 30313386 |  |  | A (0.3) | 1.33 (1.16, 1.52) | 0.0000405 | 0.99 (0.88, 1.11) | 0.847 | 1.16 (1.06, 1.27) | 0.00199 |
| rs9989761 | 2 | 132855872 |  |  | C (0.23) | 1.37 (1.18, 1.59) | 0.000041 | 1.02 (0.89, 1.16) | 0.809 | 1.15 (1.04, 1.27) | 0.00788 |
| rs10244338 | 7 | 70359758 | *WBSCR17* | intron | A (0.21) | 1.38 (1.18, 1.60) | 0.0000418 | 0.88 (0.76, 1.01) | 0.0598 | 1.12 (1.01, 1.25) | 0.0292 |
| rs2064179 | 23 | 129236874 |  |  | G (0.42) | 1.34 (1.16, 1.54) | 0.0000445 | -- | -- | 1.35 (1.17, 1.55) | 0.0000344 |
| rs2301343 | 2 | 40533653 |  |  | C (0.26) | 0.74 (0.64, 0.85) | 0.0000452 | 1.09 (0.94, 1.27) | 0.25 | 0.90 (0.81, 1.01) | 0.0623 |
| rs16974980 | 16 | 83530064 |  |  | G (0.3) | 1.32 (1.16, 1.53) | 0.0000466 | 0.88 (0.76, 1.01) | 0.0735 | 1.32 (1.16, 1.51) | 0.0000538 |
| rs589104 | 11 | 105312982 | *GRIA4* | intron | A (0.46) | 0.77 (0.68, 0.87) | 0.0000466 | 1.07 (0.95, 1.19) | 0.269 | 0.96 (0.88, 1.05) | 0.385 |
| rs2714068 | 11 | 122904751 | *GRAMD1B* | intron | A (0.4) | 1.31 (1.15, 1.49) | 0.0000524 | 0.99 (0.85, 1.14) | 0.855 | 1.30 (1.14, 1.48) | 0.0000631 |
| rs1939153 | 11 | 105256936 | *GRIA4* | intron | A (0.49) | 0.77 (0.68, 0.87) | 0.0000552 | 1.01 (0.89, 1.14) | 0.901 | 0.77 (0.67, 0.87) | 0.0000529 |
| rs256335 | 19 | 39007736 |  |  | A (0.47) | 0.77 (0.68, 0.87) | 0.0000575 | 1.30 (1.10, 1.53) | 0.00157 | 0.77 (0.68, 0.88) | 0.0000889 |
| rs3798267 | 6 | 46058786 | *CLIC5* | intron | A (0.3) | 1.33 (1.16, 1.52) | 0.000061 | 0.95 (0.84, 1.07) | 0.414 | 1.13 (1.03, 1.24) | 0.0109 |
| rs2844780 | 6 | 30312505 |  |  | A (0.3) | 1.32 (1.15, 1.51) | 0.0000668 | 0.99 (0.88, 1.12) | 0.921 | 1.15 (1.05, 1.26) | 0.00230 |
| rs11842468 | 13 | 55833285 |  |  | A (0.07) | 1.64 (1.28, 2.09) | 0.000069 | -- | -- | 1.33 (1.13, 1.57) | 0.000697 |
| rs11061995 | 12 | 1815303 | *CACNA2D4, LRTM2* | intron, 3'-UTR | A (0.18) | 1.39 (1.18, 1.64) | 0.0000772 | -- | -- | 1.34 (1.15, 1.57) | 0.000245 |
| rs2529489 | 7 | 110947132 | *IMMP2L* | intron | G (0.38) | 0.77 (0.68, 0.88) | 0.0000803 | -- | -- | 0.90 (0.83, 0.99) | 0.0231 |
| rs1331501 | 9 | 92432152 | *DIRAS2* | intron | G (0.28) | 1.32 (1.15, 1.52) | 0.0000869 | -- | -- | 1.11 (1.01, 1.22) | 0.0356 |
| rs4234232 | 3 | 34051850 |  |  | A (0.32) | 0.76 (0.66, 0.87) | 0.0000903 | -- | -- | 0.76 (0.66, 0.87) | 0.0000622 |
| rs1393576 | 4 | 160707662 |  |  | G (0.43) | 1.29 (1.14, 1.47) | 0.0000911 | -- | -- | 1.16 (1.06, 1.26) | 0.000920 |

* OR = Odds Ratio *** Freq. = Frequency

** CI = Confidence Interval **** Gene Annotation using SNPper database (Riva and Kohane, 2002) [1]
