# Supplementary material for: Dementia Revealed: Novel Chromosome 6 Locus for Late-Onset Alzheimer Disease Provides Genetic Evidence for Folate-Pathway Abnormalities
Source: PLoS Genet. 2010 Sep 23;6(9):e1001130. doi: 10.1371/journal.pgen.1001130 (PMC2944795; doi:10.1371/journal.pgen.1001130)
Supplement: Table S2 — Genotyped and imputed single nucleotide polymorphisms (SNPs) demonstrating association with late-onset Alzheimer Disease at P<10−4 in association tests adjusting for covariates from principal components capturing population substructure, evaluated in the Discovery genome-wide association study (GWAS) dataset of 931 independent cases and 1,104 independent cognitively normal controls, in the Replication GWAS dataset of 1,242 independent cases and 1,737 independent controls, and in the Combined GWAS dataset of 2,174 cases and 2,181 controls. (0.30 MB DOC) [file pgen.1001130.s004.doc]

|  |  |  |  |  |  | **Discovery GWAS** | | **Replication GWAS** | | **Combined GWAS** | |
| --- | --- | --- | --- | --- | --- | --- | --- | --- | --- | --- | --- |
| **SNP** | **Chr** | **Location** | **Gene****** | **Function****** | **Minor Allele (Freq.***)** | **OR* (95% CI**)** | **P** | **OR* (95% CI**)** | **P** | **OR* (95% CI**)** | **P** |
| rs2075650 | 19 | 50087459 | *TOMM40* | Intron (boundary) | G (0.2) | 2.95 (2.50, 3.50) | 1.62×10^-36 | 6.87 (4.23, 11.1) | 6.03×10^-15 | 3.29 (2.82, 3.83) | 2.46×10^-52 |
| rs157582 | 19 | 50088059 | *TOMM40* | Intron (boundary) | A (0.28) | 2.45 (2.11, 2.84) | 1.18×10^-31 | 5.70 (3.72, 8.72) | 1.31×10^-15 | 2.72 (2.37, 3.13) | 3.55×10^-45 |
| rs405509 | 19 | 50100676 | *APOE* | Promoter | C (0.48) | 0.62 (0.55, 0.70) | 1.49×10^-13 | 0.80 (0.54, 1.17) | 0.253 | 0.63 (0.56, 0.71) | 8.14×10^-14 |
| rs8106922 | 19 | 50093506 | *TOMM40* | Intron | G (0.36) | 0.62 (0.54, 0.71) | 3.34×10^-12 | 1.17 (0.81, 1.70) | 0.408 | 0.67 (0.59, 0.76) | 3.05×10^-10 |
| rs157580 | 19 | 50087106 | *TOMM40* | Intron | G (0.35) | 0.66 (0.57, 0.75) | 1.32×10^-9 | 0.41 (0.28, 0.60) | 5.42×10^-6 | 0.63 (0.55, 0.71) | 7.78×10^-13 |
| rs439401 | 19 | 50106291 | *APOE* | Downstream | A (0.34) | 0.66 (0.58, 0.76) | 2.45×10^-9 | 0.35 (0.23, 0.55) | 3.15×10^-6 | 0.63 (0.55, 0.72) | 3.80×10^-12 |
| rs11754661 | 6 | 151248771 | *MTHFD1L* | Intron | A (0.07) | 2.03 (1.57, 2.62) | 4.97×10^-8 | 2.23 (1.24, 4.01) | 0.00777 | 2.10 (1.67, 2.64) | 1.90×10^-10 |
| rs6859 | 19 | 50073874 | *PVRL2* | 3' UTR | A (0.46) | 1.41 (1.24, 1.60) | 1.05×10^-7 | 1.73 (1.35, 2.22) | 1.64×10^-5 | 1.55 (1.39, 1.73) | 7.33×10^-15 |
| rs10402271 | 19 | 50021054 | *BCAM* | Downstream | C (0.36) | 1.39 (1.22, 1.59) | 6.91×10^-7 | 1.16 (1.03, 1.31) | 0.0178 | 1.26 (1.16, 1.38) | 2.14×10^-7 |
| rs2252811 | 10 | 49862126 |  |  | C (0.46) | 0.73 (0.65, 0.83) | 1.55×10^-6 | 0.95 (0.83, 1.08) | 0.410 | 0.84 (0.77, 0.92) | 0.000111 |
| rs6773562 | 3 | 23633034 |  |  | C (0.13) | 1.61 (1.32, 1.97) | 2.88×10^-6 | 0.83 (0.70, 0.99) | 0.0361 | 1.09 (0.96, 1.24) | 0.174 |
| rs7699794 | 4 | 138565789 |  |  | A (0.49) | 0.74 (0.65, 0.84) | 4.34×10^-6 | 0.92 (0.81, 1.05) | 0.203 | 0.83 (0.76, 0.91) | 4.37×10^-5 |
| rs10005776 | 4 | 111235463 | *ELOVL6* | Intron | G (0.45) | 1.37 (1.20, 1.57) | 4.48×10^-6 | 0.96 (0.85, 1.09) | 0.548 | 1.12 (1.02, 1.22) | 0.0142 |
| rs12645160 | 4 | 160688870 |  |  | A (0.42) | 1.35 (1.19, 1.54) | 4.96×10^-6 | 1.03 (0.91, 1.16) | 0.685 | 1.18 (1.08, 1.28) | 0.000303 |
| rs12047155 | 1 | 50189517 |  |  | A (0.42) | 1.34 (1.18, 1.53) | 5.42×10^-6 | 0.92 (0.82, 1.04) | 0.204 | 1.11 (1.02, 1.21) | 0.0218 |
| rs6509916 | 19 | 60254214 | *RDH13* | Intron | G (0.46) | 1.34 (1.18, 1.52) | 6.85×10^-6 | 0.90 (0.79, 1.02) | 0.101 | 1.10 (1.01, 1.20) | 0.0334 |
| rs509512 | 11 | 105350133 | *GRIA4* | Intron | C (0.43) | 0.75 (0.66, 0.85) | 7.54×10^-6 | 1.13 (1.01, 1.27) | 0.0410 | 0.94 (0.86, 1.02) | 0.133 |
| rs679670 | 6 | 138179244 |  |  | G (0.37) | 0.74 (0.65, 0.85) | 1.03×10^-5 | 1.22 (0.99, 1.51) | 0.0589 | 0.87 (0.78, 0.97) | 0.0160 |
| rs9660278 | 1 | 50272772 |  |  | C (0.42) | 1.33 (1.17, 1.51) | 1.06×10^-5 | -- | -- | -- | -- |
| rs4926825 | 1 | 49831163 |  |  | A (0.41) | 1.33 (1.17, 1.51) | 1.06×10^-5 | 0.93 (0.82, 1.04) | 0.205 | 1.10 (1.01, 1.20) | 0.0279 |
| rs1244096 | 12 | 123481044 | *NCOR2* | Intron (boundary) | A (0.1) | 1.61 (1.30, 1.99) | 1.20×10^-5 | 1.14 (0.82, 1.59) | 0.423 | 1.49 (1.25, 1.78) | 8.00×10^-6 |
| rs799419 | 6 | 138180548 |  |  | G (0.37) | 0.74 (0.65, 0.85) | 1.28×10^-5 | 1.23 (1.00, 1.53) | 0.0511 | 0.87 (0.78, 0.98) | 0.0176 |
| rs669397 | 11 | 105351597 | *GRIA4* | Intron | G (0.43) | 0.75 (0.66, 0.86) | 1.31×10^-5 | 1.13 (1.00, 1.27) | 0.0495 | 0.93 (0.86, 1.02) | 0.123 |
| rs10225470 | 7 | 54155997 |  |  | A (0.15) | 0.68 (0.57, 0.81) | 1.37×10^-5 | 0.95 (0.83, 1.09) | 0.474 | 0.81 (0.73, 0.91) | 0.000161 |
| rs8074294 | 17 | 61902137 | *PRKCA* | Intron | G (0.39) | 0.75 (0.66, 0.85) | 1.41×10^-5 | 1.16 (1.03, 1.31) | 0.0184 | 0.94 (0.86, 1.03) | 0.198 |
| rs1167272 | 1 | 49658574 |  |  | A (0.31) | 1.36 (1.18, 1.56) | 1.58×10^-5 | 0.99 (0.87, 1.13) | 0.864 | 1.14 (1.04, 1.26) | 0.00491 |
| rs17379721 | 1 | 50049596 |  |  | A (0.41) | 1.32 (1.16, 1.50) | 1.67×10^-5 | 0.93 (0.82, 1.04) | 0.211 | 1.10 (1.01, 1.19) | 0.0374 |
| rs11025237 | 11 | 19726288 | *NAV2* | Intron | A (0.2) | 1.42 (1.21, 1.67) | 1.71×10^-5 | 1.01 (0.88, 1.17) | 0.873 | 1.17 (1.05, 1.30) | 0.00377 |
| rs7091819 | 10 | 26028836 |  |  | C (0.24) | 1.39 (1.19, 1.61) | 1.71×10^-5 | 1.02 (0.89, 1.17) | 0.808 | 1.18 (1.07, 1.30) | 0.00109 |
| rs12083887 | 1 | 118683212 |  |  | A (0.4) | 0.75 (0.65, 0.85) | 1.74×10^-5 | 0.94 (0.83, 1.07) | 0.359 | 0.84 (0.77, 0.92) | 0.000185 |
| rs8113032 | 19 | 60245950 | *RDH13* | Downstream | A (0.41) | 1.32 (1.16, 1.50) | 1.80×10^-5 | 0.88 (0.78, 1.00) | 0.0467 | 1.07 (0.98, 1.17) | 0.110 |
| rs12135821 | 1 | 118744972 |  |  | A (0.47) | 1.33 (1.17, 1.51) | 1.81×10^-5 | 1.11 (0.98, 1.27) | 0.107 | 1.20 (1.09, 1.31) | 9.13×10^-5 |
| rs11131099 | 3 | 823802 |  |  | G (0.37) | 0.75 (0.65, 0.85) | 1.85×10^-5 | 0.94 (0.83, 1.07) | 0.368 | 0.84 (0.77, 0.92) | 0.000222 |
| rs1893953 | 4 | 160693858 |  |  | C (0.47) | 1.32 (1.16, 1.50) | 1.85×10^-5 | 1.04 (0.92, 1.18) | 0.494 | 1.17 (1.08, 1.28) | 0.000308 |
| rs4676049 | 2 | 109001689 |  |  | A (0.08) | 1.62 (1.30, 2.03) | 1.98×10^-5 | 1.32 (0.75, 2.32) | 0.342 | 1.76 (1.44, 2.15) | 4.31×10^-8 |
| rs7303876 | 12 | 58421479 | *SLC16A7* | Intron | G (0.26) | 0.72 (0.63, 0.84) | 1.98×10^-5 | 0.67 (0.41, 1.11) | 0.118 | 0.72 (0.62, 0.83) | 4.87×10^-7 |
| rs1856297 | 1 | 49810849 |  |  | A (0.41) | 1.32 (1.16, 1.50) | 2.14×10^-5 | 0.93 (0.82, 1.04) | 0.205 | 1.10 (1.01, 1.20) | 0.0369 |
| rs4388744 | 1 | 50294191 |  |  | A (0.32) | 1.34 (1.17, 1.53) | 2.15×10^-5 | -- | -- | -- | -- |
| rs11100238 | 4 | 160696507 |  |  | G (0.47) | 1.32 (1.16, 1.50) | 2.16×10^-5 | -- | -- | -- | -- |
| rs4489606 | 1 | 50287140 |  |  | G (0.32) | 1.34 (1.17, 1.53) | 2.23×10^-5 | 0.98 (0.86, 1.12) | 0.783 | 1.14 (1.04, 1.25) | 0.00531 |
| rs12255607 | 10 | 20181408 | *PLXDC2* | Intron | G (0.17) | 1.46 (1.23, 1.74) | 2.28×10^-5 | 0.86 (0.70, 1.06) | 0.170 | 1.21 (1.06, 1.38) | 0.00432 |
| rs4926814 | 1 | 49729723 |  |  | A (0.41) | 1.32 (1.16, 1.50) | 2.34×10^-5 | 0.91 (0.81, 1.03) | 0.138 | 1.09 (1.00, 1.19) | 0.0465 |
| rs4926547 | 1 | 50319397 |  |  | G (0.32) | 1.34 (1.17, 1.53) | 2.43×10^-5 | 0.98 (0.86, 1.11) | 0.706 | 1.13 (1.03, 1.24) | 0.00764 |
| rs11038913 | 11 | 46516306 | *FLJ20294* | Intron | G (0.1) | 1.60 (1.28, 1.98) | 2.48×10^-5 | 0.77 (0.58, 1.02) | 0.0716 | 1.28 (1.08, 1.50) | 0.00390 |
| rs1415985 | 1 | 49703336 |  |  | A (0.41) | 1.31 (1.16, 1.49) | 2.68×10^-5 | -- | -- | -- | -- |
| rs2975139 | 12 | 16393084 | *MGST1* | Intron | C (0.26) | 0.74 (0.64, 0.85) | 2.78×10^-5 | 0.91 (0.57, 1.44) | 0.680 | 0.85 (0.75, 0.96) | 0.00723 |
| rs17034806 | 2 | 109002337 |  |  | G (0.08) | 1.61 (1.29, 2.01) | 2.79×10^-5 | 1.36 (0.77, 2.42) | 0.289 | 1.75 (1.43, 2.15) | 5.14×10^-8 |
| rs2305543 | 19 | 60251527 | *RDH13* | Synonymous Coding (L143L) | A (0.25) | 1.36 (1.18, 1.56) | 2.81×10^-5 | 0.95 (0.82, 1.11) | 0.524 | 1.18 (1.06, 1.30) | 0.00205 |
| rs11720720 | 3 | 65794510 | *MAGI1* | Intron | A (0.08) | 1.71 (1.33, 2.20) | 2.82×10^-5 | -- | -- | -- | -- |
| rs2529491 | 7 | 110959870 | *IMMP2L* | Intron | A (0.32) | 0.74 (0.65, 0.85) | 2.92×10^-5 | 0.92 (0.80, 1.05) | 0.225 | 0.85 (0.77, 0.94) | 0.00103 |
| rs1360873 | 13 | 63489710 |  |  | A (0.18) | 0.70 (0.60, 0.83) | 3.02×10^-5 | 1.03 (0.88, 1.20) | 0.733 | 0.86 (0.77, 0.96) | 0.00879 |
| rs6957883 | 7 | 147870112 |  |  | G (0.38) | 0.75 (0.66, 0.86) | 3.15×10^-5 | 0.85 (0.75, 0.96) | 0.0113 | 0.81 (0.74, 0.88) | 2.29×10^-6 |
| rs1538981 | 10 | 31451361 |  |  | G (0.5) | 1.32 (1.16, 1.51) | 3.34×10^-5 | 0.95 (0.84, 1.07) | 0.399 | 1.10 (1.01, 1.20) | 0.0325 |
| rs1185222 | 1 | 49731548 |  |  | A (0.31) | 1.33 (1.16, 1.52) | 3.36×10^-5 | -- | -- | -- | -- |
| rs1727987 | 1 | 49761808 |  |  | A (0.31) | 1.33 (1.16, 1.52) | 3.36×10^-5 | 0.99 (0.87, 1.12) | 0.819 | 1.14 (1.04, 1.25) | 0.00657 |
| rs10888665 | 1 | 49911493 |  |  | A (0.31) | 1.33 (1.16, 1.52) | 3.36×10^-5 | 0.97 (0.86, 1.11) | 0.696 | 1.13 (1.03, 1.24) | 0.00799 |
| rs2050876 | 10 | 31093734 |  |  | T (0.23) | 0.72 (0.62, 0.84) | 3.36×10^-5 | 1.34 (1.12, 1.60) | 0.00148 | 0.95 (0.85, 1.07) | 0.387 |
| rs1167262 | 1 | 49646567 |  |  | A (0.31) | 1.33 (1.16, 1.52) | 3.43×10^-5 | 0.98 (0.87, 1.12) | 0.807 | 1.14 (1.04, 1.25) | 0.00605 |
| rs10888679 | 1 | 50338853 | *ELAVL4* | Promoter | G (0.31) | 1.33 (1.16, 1.53) | 3.43×10^-5 | 0.97 (0.85, 1.10) | 0.598 | 1.13 (1.03, 1.24) | 0.00930 |
| rs1891667 | 1 | 49867972 |  |  | A (0.31) | 1.33 (1.16, 1.52) | 3.50×10^-5 | 0.98 (0.86, 1.12) | 0.792 | 1.14 (1.04, 1.25) | 0.00637 |
| rs2000886 | 4 | 160740930 |  |  | A (0.47) | 0.76 (0.67, 0.87) | 3.54×10^-5 | 1.01 (0.89, 1.14) | 0.890 | 0.88 (0.81, 0.97) | 0.00623 |
| rs1112687 | 1 | 49841291 |  |  | A (0.31) | 1.33 (1.16, 1.52) | 3.57×10^-5 | 0.98 (0.86, 1.11) | 0.740 | 1.13 (1.03, 1.24) | 0.00746 |
| rs7019702 | 9 | 132897543 | *GFI1B* | Downstream | C (0.18) | 1.43 (1.21, 1.69) | 3.58×10^-5 | 1.46 (1.05, 2.02) | 0.0240 | 1.42 (1.22, 1.64) | 3.45×10^-6 |
| rs3957 | 1 | 49630498 |  |  | C (0.31) | 1.33 (1.16, 1.52) | 3.74×10^-5 | -- | -- | -- | -- |
| rs9989761 | 2 | 132855872 |  |  | C (0.23) | 1.37 (1.18, 1.59) | 3.74×10^-5 | 0.98 (0.85, 1.13) | 0.784 | 1.15 (1.04, 1.27) | 0.00788 |
| rs12049328 | 1 | 49463210 |  |  | T (0.4) | 1.31 (1.15, 1.49) | 3.75×10^-5 | -- | -- | -- | -- |
| rs1343161 | 1 | 49883437 |  |  | G (0.31) | 1.33 (1.16, 1.52) | 3.80×10^-5 | 0.97 (0.85, 1.10) | 0.630 | 1.13 (1.03, 1.24) | 0.0112 |
| rs16974980 | 16 | 83530064 |  |  | G (0.31) | 1.33 (1.16, 1.52) | 3.81×10^-5 | 0.85 (0.72, 0.99) | 0.0386 | 1.10 (1.00, 1.22) | 0.0571 |
| rs13151952 | 4 | 138539922 |  |  | A (0.45) | 0.76 (0.67, 0.87) | 3.85×10^-5 | 0.98 (0.87, 1.10) | 0.689 | 0.88 (0.81, 0.96) | 0.00379 |
| rs1713417 | 14 | 19933651 | *TEP1* | Intron (boundary) | A (0.07) | 1.65 (1.30, 2.09) | 3.87×10^-5 | 1.02 (0.82, 1.26) | 0.883 | 1.24 (1.06, 1.45) | 0.00647 |
| rs6693294 | 1 | 49651709 |  |  | A (0.31) | 1.32 (1.16, 1.51) | 3.89×10^-5 | 0.98 (0.85, 1.13) | 0.784 | 1.16 (1.06, 1.28) | 0.00196 |
| rs4820297 | 22 | 36435927 | *TRIOBP* | Intron | G (0.42) | 1.33 (1.16, 1.52) | 3.92×10^-5 | 1.06 (0.93, 1.21) | 0.386 | 1.18 (1.08, 1.30) | 0.000396 |
| rs1577969 | 1 | 49637882 |  |  | T (0.31) | 1.32 (1.16, 1.51) | 3.96×10^-5 | -- | -- | -- | -- |
| rs10736388 | 1 | 50108115 |  |  | A (0.31) | 1.33 (1.16, 1.52) | 3.97×10^-5 | 0.96 (0.85, 1.10) | 0.589 | 1.13 (1.03, 1.24) | 0.0122 |
| rs1112368 | 1 | 49932177 |  |  | G (0.31) | 1.32 (1.16, 1.51) | 3.99×10^-5 | -- | -- | -- | -- |
| rs7530169 | 1 | 49941178 |  |  | A (0.31) | 1.32 (1.16, 1.51) | 3.99×10^-5 | 0.97 (0.86, 1.11) | 0.679 | 1.13 (1.03, 1.24) | 0.00921 |
| rs5930403 | 23 | 129318208 | *SLC25A14* | Intron | T (0.44) | 0.74 (0.65, 0.86) | 4.00×10^-5 | -- | -- | -- | -- |
| rs1179484 | 1 | 49681231 |  |  | C (0.31) | 1.32 (1.16, 1.51) | 4.04×10^-5 | 1.00 (0.87, 1.14) | 0.960 | 1.15 (1.04, 1.26) | 0.00404 |
| rs1167270 | 1 | 49685647 |  |  | A (0.31) | 1.32 (1.16, 1.51) | 4.04×10^-5 | 0.99 (0.87, 1.12) | 0.850 | 1.14 (1.04, 1.25) | 0.00569 |
| rs5977248 | 23 | 129329168 | *SLC25A14* | Intron | A (0.44) | 0.74 (0.65, 0.86) | 4.04×10^-5 | -- | -- | -- | -- |
| rs1494462 | 1 | 49568744 |  |  | A (0.4) | 1.31 (1.15, 1.49) | 4.15×10^-5 | 0.94 (0.83, 1.06) | 0.308 | 1.10 (1.01, 1.20) | 0.0308 |
| rs2301343 | 2 | 40533653 |  |  | C (0.26) | 0.74 (0.64, 0.85) | 4.16×10^-5 | 1.07 (0.91, 1.26) | 0.432 | 0.90 (0.81, 1.01) | 0.0623 |
| rs2064179 | 23 | 129236874 | *SUHW3* | Promoter | G (0.42) | 1.34 (1.16, 1.54) | 4.34×10^-5 | -- | -- | -- | -- |
| rs2846215 | 11 | 105376028 | *KIAA1826* | Downstream | G (0.43) | 0.76 (0.66, 0.87) | 4.34×10^-5 | 1.11 (0.99, 1.25) | 0.0808 | 0.94 (0.86, 1.03) | 0.162 |
| rs4926812 | 1 | 49586400 |  |  | A (0.31) | 1.32 (1.16, 1.51) | 4.35×10^-5 | -- | -- | -- | -- |
| rs1338214 | 1 | 49596084 |  |  | C (0.31) | 1.32 (1.16, 1.51) | 4.39×10^-5 | 0.98 (0.86, 1.11) | 0.732 | 1.13 (1.03, 1.24) | 0.00909 |
| rs10259067 | 7 | 95069392 | *PDK4* | Promoter | A (0.05) | 1.86 (1.38, 2.51) | 4.39×10^-5 | 1.23 (0.76, 1.98) | 0.401 | 1.62 (1.28, 2.04) | 4.85×10^-5 |
| rs10244338 | 7 | 70359758 | *WBSCR17* | Intron | A (0.21) | 1.37 (1.18, 1.60) | 4.52×10^-5 | 0.89 (0.76, 1.03) | 0.111 | 1.12 (1.01, 1.25) | 0.0292 |
| rs6588362 | 1 | 50066939 |  |  | T (0.31) | 1.32 (1.16, 1.51) | 4.59×10^-5 | -- | -- | -- | -- |
| rs10888669 | 1 | 50022860 |  |  | T (0.37) | 1.31 (1.15, 1.48) | 4.65×10^-5 | 0.98 (0.87, 1.11) | 0.759 | 1.12 (1.03, 1.23) | 0.00922 |
| rs2787693 | 1 | 49688435 |  |  | C (0.31) | 1.32 (1.16, 1.51) | 4.65×10^-5 | 0.99 (0.87, 1.13) | 0.893 | 1.14 (1.04, 1.25) | 0.00597 |
| rs589104 | 11 | 105312982 | *GRIA4* | Intron | A (0.46) | 0.77 (0.68, 0.87) | 4.72×10^-5 | 1.17 (1.04, 1.32) | 0.0105 | 0.96 (0.88, 1.05) | 0.385 |
| rs3092217 | 20 | 39813482 |  |  | G (0.24) | 1.36 (1.17, 1.57) | 4.73×10^-5 | 0.87 (0.75, 1.01) | 0.0673 | 1.10 (0.99, 1.22) | 0.0756 |
| rs4926545 | 1 | 50152063 |  |  | A (0.31) | 1.32 (1.16, 1.51) | 4.76×10^-5 | 0.98 (0.86, 1.12) | 0.778 | 1.13 (1.03, 1.24) | 0.00828 |
| rs6697839 | 1 | 50163925 |  |  | A (0.31) | 1.32 (1.16, 1.51) | 4.76×10^-5 | 0.98 (0.86, 1.12) | 0.778 | 1.13 (1.03, 1.24) | 0.00828 |
| rs10788924 | 1 | 50168618 |  |  | A (0.31) | 1.32 (1.16, 1.51) | 4.76×10^-5 | 0.98 (0.86, 1.12) | 0.778 | 1.13 (1.03, 1.24) | 0.00828 |
| rs11603669 | 11 | 134212161 |  |  | A (0.18) | 1.41 (1.19, 1.65) | 4.78×10^-5 | 1.09 (0.94, 1.27) | 0.251 | 1.20 (1.07, 1.33) | 0.00126 |
| rs5932752 | 23 | 129334460 | *SLC25A14* | Intron | A (0.44) | 0.75 (0.65, 0.86) | 4.81×10^-5 | -- | -- | -- | -- |
| rs12036551 | 1 | 49555928 |  |  | A (0.4) | 1.30 (1.15, 1.48) | 4.81×10^-5 | 0.94 (0.83, 1.06) | 0.2873 | 1.10 (1.01, 1.20) | 0.0322 |
| rs7795083 | 7 | 70376454 | *WBSCR17* | Intron | A (0.21) | 1.38 (1.18, 1.61) | 4.85×10^-5 | 0.87 (0.75, 1.02) | 0.0864 | 1.13 (1.01, 1.25) | 0.0329 |
| rs3798267 | 6 | 46058786 | *CLIC5* | Intron | A (0.3) | 1.33 (1.16, 1.53) | 4.98×10^-5 | 0.95 (0.83, 1.08) | 0.445 | 1.13 (1.03, 1.24) | 0.0109 |
| rs6029791 | 20 | 39794198 |  |  | T (0.16) | 1.44 (1.21, 1.72) | 5.03×10^-5 | -- | -- | -- | -- |
| rs10424969 | 19 | 60258324 | *RDH13* | Intron | C (0.12) | 0.64 (0.52, 0.80) | 5.15×10^-5 | 1.06 (0.88, 1.27) | 0.537 | 0.83 (0.72, 0.95) | 0.00790 |
| rs6693846 | 1 | 50087939 |  |  | A (0.31) | 1.32 (1.15, 1.51) | 5.42×10^-5 | 0.98 (0.86, 1.12) | 0.795 | 1.13 (1.03, 1.24) | 0.00920 |
| rs7544728 | 1 | 50112236 |  |  | A (0.31) | 1.32 (1.15, 1.51) | 5.42×10^-5 | 0.98 (0.86, 1.12) | 0.791 | 1.13 (1.03, 1.24) | 0.00854 |
| rs2832594 | 21 | 30388188 |  |  | G (0.31) | 1.33 (1.16, 1.52) | 5.51×10^-5 | 0.96 (0.85, 1.08) | 0.490 | 1.12 (1.02, 1.22) | 0.0180 |
| rs1939153 | 11 | 105256936 | *GRIA4* | Intron | A (0.49) | 0.77 (0.68, 0.87) | 5.54×10^-5 | 0.90 (0.78, 1.04) | 0.139 | 1.11 (1.01, 1.21) | 0.0360 |
| rs11842468 | 13 | 55833285 |  |  | A (0.07) | 1.65 (1.29, 2.10) | 5.60×10^-5 | 1.08 (0.86, 1.37) | 0.515 | 1.33 (1.13, 1.57) | 0.000697 |
| rs12713404 | 2 | 59860209 |  |  | C (0.37) | 1.31 (1.15, 1.50) | 5.66×10^-5 | 0.94 (0.83, 1.06) | 0.307 | 1.09 (1.00, 1.19) | 0.0573 |
| rs12743369 | 1 | 50337003 |  |  | A (0.4) | 1.30 (1.15, 1.49) | 5.80×10^-5 | 0.92 (0.81, 1.04) | 0.169 | 1.09 (0.99, 1.18) | 0.0700 |
| rs256335 | 19 | 39007736 |  |  | A (0.47) | 0.77 (0.68, 0.87) | 5.81×10^-5 | 1.28 (1.06, 1.54) | 0.0109 | 0.92 (0.82, 1.02) | 0.102 |
| rs7540194 | 1 | 49563736 |  |  | C (0.31) | 1.32 (1.15, 1.51) | 5.93×10^-5 | -- | -- | -- | -- |
| rs2714068 | 11 | 122904751 |  |  | A (0.4) | 1.30 (1.15, 1.48) | 5.96×10^-5 | 1.06 (0.89, 1.26) | 0.529 | 1.21 (1.09, 1.34) | 0.000280 |
| rs4806636 | 19 | 60240370 | *GP6* | Intron | A (0.49) | 1.30 (1.14, 1.48) | 6.23×10^-5 | -- | -- | -- | -- |
| rs3899856 | 1 | 50016441 |  |  | A (0.37) | 1.30 (1.14, 1.48) | 6.30×10^-5 | 0.97 (0.86, 1.10) | 0.677 | 1.12 (1.03, 1.22) | 0.0120 |
| rs6695041 | 1 | 50018096 |  |  | A (0.37) | 1.30 (1.14, 1.48) | 6.30×10^-5 | 0.97 (0.86, 1.10) | 0.677 | 1.12 (1.03, 1.22) | 0.0120 |
| rs5932738 | 23 | 129242314 |  |  | A (0.41) | 1.33 (1.16, 1.53) | 6.42×10^-5 | -- | -- | -- | -- |
| rs1654431 | 19 | 60241570 | *GP6* | Promoter | A (0.49) | 1.30 (1.14, 1.48) | 6.54×10^-5 | 1.03 (0.91, 1.16) | 0.661 | 1.12 (1.02, 1.22) | 0.0154 |
| rs241472 | 1 | 49544440 |  |  | G (0.31) | 1.31 (1.15, 1.50) | 6.66×10^-5 | 0.95 (0.84, 1.09) | 0.464 | 1.11 (1.01, 1.22) | 0.0245 |
| rs11061995 | 12 | 1815303 | *LRTM2* | 3' UTR | A (0.18) | 1.40 (1.18, 1.64) | 6.89×10^-5 | 1.02 (0.61, 1.72) | 0.936 | 1.34 (1.15, 1.57) | 0.000245 |
| rs2529489 | 7 | 110947132 | *IMMP2L* | Intron | G (0.38) | 0.77 (0.68, 0.88) | 7.22×10^-5 | 1.00 (0.89, 1.13) | 0.990 | 0.90 (0.83, 0.99) | 0.0231 |
| rs12976416 | 19 | 34053326 |  |  | C (0.25) | 0.74 (0.64, 0.86) | 7.39×10^-5 | 1.06 (0.92, 1.23) | 0.435 | 0.90 (0.81, 1.00) | 0.0504 |
| rs1010978 | 23 | 129307141 | *SLC25A14* | Intron | G (0.44) | 0.75 (0.65, 0.87) | 7.45×10^-5 | -- | -- | -- | -- |
| rs6449493 | 5 | 60082253 | *ELOVL7* | Downstream | A (0.28) | 1.32 (1.15, 1.51) | 7.48×10^-5 | 1.02 (0.81, 1.28) | 0.879 | 1.18 (1.05, 1.31) | 0.00417 |
| rs4830186 | 23 | 129340004 | *GPR119* | Downstream | G (0.44) | 0.75 (0.65, 0.87) | 7.61×10^-5 | -- | -- | -- | -- |
| rs4830187 | 23 | 129340424 | *GPR119* | Downstream | A (0.44) | 0.75 (0.65, 0.87) | 7.85×10^-5 | -- | -- | -- | -- |
| rs4582848 | 1 | 50084787 |  |  | A (0.31) | 1.31 (1.14, 1.49) | 7.97×10^-5 | 0.97 (0.85, 1.10) | 0.618 | 1.12 (1.02, 1.23) | 0.0152 |
| rs4779542 | 15 | 29639276 | *OTUD7* | Intron | G (0.19) | 1.41 (1.19, 1.67) | 8.08×10^-5 | -- | -- | -- | -- |
| rs7574523 | 2 | 132858237 |  |  | T (0.23) | 1.36 (1.17, 1.58) | 8.21×10^-5 | -- | -- | -- | -- |
| rs869058 | 6 | 90312689 | *ANKRD6* | Intron | A (0.43) | 0.77 (0.68, 0.88) | 8.22×10^-5 | 0.95 (0.76, 1.18) | 0.652 | 0.84 (0.76, 0.94) | 0.00169 |
| rs4234232 | 3 | 34051850 |  |  | A (0.32) | 0.76 (0.66, 0.87) | 8.23×10^-5 | 0.96 (0.83, 1.10) | 0.546 | 0.85 (0.77, 0.94) | 0.00112 |
| rs10912537 | 1 | 169433156 | *FMO2* | Intron | G (0.31) | 1.32 (1.15, 1.51) | 8.33×10^-5 | 1.02 (0.90, 1.16) | 0.726 | 1.15 (1.05, 1.25) | 0.00325 |
| rs1800291 | 23 | 153811479 | *F8* | Synonymous Coding (D1260E) | C (0.21) | 0.71 (0.60, 0.84) | 8.40×10^-5 | -- | -- | -- | -- |
| rs3777599 | 6 | 46050909 | *CLIC5* | Intron | T (0.3) | 1.33 (1.16, 1.54) | 8.73×10^-5 | -- | -- | -- | -- |
| rs1393576 | 4 | 160707662 |  |  | G (0.43) | 1.29 (1.14, 1.47) | 8.95×10^-5 | 1.04 (0.92, 1.17) | 0.516 | 1.16 (1.06, 1.26) | 0.000920 |
| rs5932754 | 23 | 129342752 | *GPR119* | Downstream | A (0.44) | 0.75 (0.66, 0.87) | 9.09×10^-5 | -- | -- | -- | -- |
| rs13427042 | 2 | 132858435 |  |  | C (0.23) | 1.35 (1.16, 1.58) | 9.24×10^-5 | 0.98 (0.85, 1.13) | 0.784 | 1.14 (1.03, 1.26) | 0.0125 |
| rs10282292 | 7 | 110879714 | *IMMP2L* | Intron | G (0.38) | 0.77 (0.67, 0.88) | 9.28×10^-5 | 1.03 (0.91, 1.17) | 0.662 | 0.91 (0.83, 1.00) | 0.0465 |
| rs7080202 | 10 | 31097100 |  |  | C (0.24) | 0.74 (0.63, 0.86) | 9.38×10^-5 | 1.27 (1.06, 1.51) | 0.00797 | 0.94 (0.84, 1.05) | 0.280 |
| rs1331501 | 9 | 92432152 | *DIRAS2* | Intron | G (0.28) | 1.32 (1.15, 1.52) | 9.55×10^-5 | 0.95 (0.83, 1.08) | 0.451 | 1.11 (1.01, 1.22) | 0.0356 |
| rs867991 | 10 | 31429271 |  |  | C (0.48) | 0.77 (0.68, 0.88) | 9.59×10^-5 | 1.01 (0.90, 1.14) | 0.856 | 0.90 (0.82, 0.98) | 0.0152 |
| rs2292585 | 10 | 49856323 |  |  | G (0.49) | 1.29 (1.13, 1.46) | 9.60×10^-5 | 0.97 (0.86, 1.11) | 0.6919 | 0.88 (0.81, 0.96) | 0.00530 |
| rs4830188 | 23 | 129342104 | *GPR119* | Downstream | A (0.44) | 0.76 (0.66, 0.87) | 9.60×10^-5 | -- | -- | -- | -- |
| rs1998587 | 9 | 78268523 | *GCNT1* | Intron | C (0.12) | 1.51 (1.23, 1.85) | 9.71×10^-5 | 0.99 (0.83, 1.18) | 0.8887 | 1.15 (1.01, 1.31) | 0.0356 |
| rs9544105 | 13 | 75456154 |  |  | G (0.27) | 1.32 (1.15, 1.53) | 9.79×10^-5 | 1.01 (0.88, 1.16) | 0.8997 | 1.15 (1.05, 1.27) | 0.00373 |
| rs12610605 | 19 | 50062678 | *PVRL2* | Intron | A (0.17) | 0.71 (0.60, 0.85) | 9.83×10^-5 | 0.53 (0.40, 0.69) | 4.64×10^-6 | 0.69 (0.60, 0.79) | 1.59×10^-7 |

* OR = Odds Ratio *** Freq. = Frequency

** CI = Confidence Interval **** Gene Annotation using SNPper database (Riva and Kohane, 2002) [1]
