# Supplementary material for: Dementia Revealed: Novel Chromosome 6 Locus for Late-Onset Alzheimer Disease Provides Genetic Evidence for Folate-Pathway Abnormalities
Source: PLoS Genet. 2010 Sep 23;6(9):e1001130. doi: 10.1371/journal.pgen.1001130 (PMC2944795; doi:10.1371/journal.pgen.1001130)
Supplement: Table S3 — Follow-up of the strongest associations reported in the Beecham, et al.(2009) [18] GWAS of late-onset Alzheimer Disease. 32 single nucleotide polymorphisms (SNPs) demonstrating the strongest association with late-onset Alzheimer Disease at P<10−5 in the Beecham et al. (2009) [18] GWAS of late-onset Alzheimer's Disease, tested here for association with adjustment for covariates from principal components capturing population substructure, evaluated in the Discovery genome-wide association study (GWAS) dataset of 931 independent cases and 1,104 independent cognitively normal controls. (0.06 MB DOC) [file pgen.1001130.s005.doc]

|  |  |  |  |  |  | **Beecham et al. GWAS** | **Discovery GWAS** | | |
| --- | --- | --- | --- | --- | --- | --- | --- | --- | --- |
| **SNP** | **Chr** | **Location** | **Gene***** | **Function***** | **Minor Allele**  **(Freq.)** | **P** | **OR* (95% CI**)** | | **P** |
| rs11205641 | 1 | 49957662 |  |  | A (0.42) | 8.40×10^-6 | 1.21 (1.07, 1.37) | 0.00265 | |
| rs4926831 | 1 | 50062688 |  |  | G (0.43) | 1.20×10^-5 | 1.21 (1.07, 1.37) | 0.00287 | |
| rs9659092 | 1 | 50216176 |  |  | A (0.43) | 4.50×10^-6 | 1.24 (1.09, 1.41) | 0.000888 | |
| rs11583200 | 1 | 50332407 |  |  | G (0.38) | 1.80×10^-5 | 1.27 (1.12, 1.44) | 0.000301 | |
| rs6428503 | 1 | 89356657 | *GBP2* | Intron | G (0.47) | 6.70×10^-4 | 1.10 (0.97, 1.25) | 0.124 | |
| rs11683103 | 2 | 34766354 |  |  | G (0.3) | 8.60×10^-6 | 0.78 (0.68, 0.90) | 0.000461 | |
| rs2119067 | 2 | 165835529 |  |  | G (0.23) | 4.40×10^-5 | 0.79 (0.68, 0.92) | 0.00229 | |
| rs10184275 | 2 | 165836174 |  |  | G (0.18) | 2.20×10^-5 | 0.79 (0.67, 0.93) | 0.00499 | |
| rs3846421 | 4 | 7336257 | *SORCS2* | Intron | G (0.34) | 1.20×10^-4 | 0.88 (0.77, 1.01) | 0.0670 | |
| rs12639920 | 4 | 42107444 | *ATP8A1* | Downstream | A (0.28) | 4.90×10^-5 | 1.28 (1.11, 1.47) | 0.000550 | |
| rs3807031 | 6 | 30141863 | *PPP1R11* | Promoter | A (0.2) | 1.20×10^-5 | 1.30 (1.11, 1.52) | 0.00119 | |
| rs929156 | 6 | 30247678 | *TRIM15* | Intron | A (0.24) | 1.70×10^-5 | 1.38 (1.19, 1.60) | 0.0000257 | |
| rs11754661 | 6 | 151248771 | *MTHFD1L* | Intron | A (0.07) | 2.00×10^-5 | 2.03 (1.58, 2.62) | 4.70×10^-8 | |
| rs9455973 | 6 | 168325855 |  |  | A (0.1) | 4.50×10^-5 | 0.84 (0.68, 1.03) | 0.0958 | |
| rs2039461 | 9 | 20135988 |  |  | A (0.38) | 3.50×10^-5 | 1.12 (0.99, 1.28) | 0.0784 | |
| rs7893928 | 10 | 44398949 |  |  | A (0.13) | 2.30×10^-5 | 1.39 (1.16, 1.67) | 0.000422 | |
| rs10786828 | 10 | 106599890 | *SORCS3* | Intron | A (0.47) | 4.60×10^-3 | 1.10 (0.97, 1.24) | 0.155 | |
| rs11244841 | 10 | 127824556 | *ADAM12* | Intron | G (0.26) | 3.40×10^-3 | 1.08 (0.94, 1.24) | 0.282 | |
| rs3781835 | 11 | 120953464 | *SORL1* | Intron | A (0.02) | 6.20×10^-3 | 0.70 (0.44, 1.10) | 0.118 | |
| rs11610206 | 12 | 45925793 | *FAM113B* | Downstream | G (0.09) | 3.50×10^-7 | 0.68 (0.54, 0.85) | 0.000770 | |
| rs2387100 | 13 | 27324759 |  |  | G (0.27) | 3.80×10^-5 | 1.28 (1.11, 1.47) | 0.000871 | |
| rs9544105 | 13 | 75456154 |  |  | G (0.27) | 5.40×10^-6 | 1.32 (1.15, 1.52) | 0.000105 | |
| rs4555132 | 15 | 95740242 |  |  | G (0.24) | 3.10×10^-5 | 1.20 (1.04, 1.39) | 0.0139 | |
| rs1480090 | 15 | 96533184 |  |  | A (0.5) | 3.50×10^-5 | 0.86 (0.75, 0.97) | 0.0160 | |
| rs1383139 | 15 | 96535200 |  |  | G (0.49) | 3.50×10^-5 | 1.13 (1.00, 1.28) | 0.0520 | |
| rs1402627 | 18 | 4123739 |  |  | A (0.2) | 4.40×10^-5 | 0.79 (0.68, 0.93) | 0.00430 | |
| rs4459653 | 19 | 49291455 | *ZNF224* | Intron | G (0.17) | 8.00×10^-6 | 0.76 (0.64, 0.91) | 0.00190 | |
| rs4802207 | 19 | 49292217 | *ZNF224* | Intron | G (0.18) | 9.20×10^-6 | 0.76 (0.64, 0.89) | 0.00108 | |
| rs3746319 | 19 | 49304071 | *ZNF224* | Coding exon | A (0.17) | 3.00×10^-5 | 0.76 (0.65, 0.90) | 0.00162 | |
| rs2061332 | 19 | 49305501 | *ZNF224* | Downstream | A (0.17) | 3.90×10^-5 | 0.76 (0.65, 0.90) | 0.00153 | |
| rs6059244 | 20 | 29474144 |  |  | A (0.35) | 4.80×10^-5 | 1.17 (1.03, 1.34) | 0.0150 | |
| rs2180566 | 20 | 29482515 | *DEFB123* | Promoter | G (0.35) | 3.80×10^-5 | 1.18 (1.03, 1.34) | 0.0144 | |

* OR = Odds Ratio

** CI = Confidence Interval

*** Gene Annotation using SNPper database (Riva and Kohane, 2002) [1]
