# Supplementary material for: Dementia Revealed: Novel Chromosome 6 Locus for Late-Onset Alzheimer Disease Provides Genetic Evidence for Folate-Pathway Abnormalities
Source: PLoS Genet. 2010 Sep 23;6(9):e1001130. doi: 10.1371/journal.pgen.1001130 (PMC2944795; doi:10.1371/journal.pgen.1001130)
Supplement: Table S4 — Genotype frequency distributions and differences in three subsets of a GWAS dataset for SNPs with strong associations with late-onset Alzheimer Disease. Genotype counts, P-values for Hardy-Weinberg Equilibrium (HWE), and P-values for differences in genotypic distribution from a Fisher's Exact Test (FET) comparing controls in SNPs with strong associations with late-onset Alzheimer Disease in three subsets of a GWAS dataset: cognitively normal controls from the previously published Beecham et al (2009) study [18] (“Beecham et al. controls”), cognitively normal controls recruited after the Beecham et al. (2009) study (“New AD Controls”), and cogntively normal controls consented for multiple genetic studies whose recruitment was funded through the Udall Parkinson's Disease Collaboration (“Udall Controls”). (0.05 MB DOC) [file pgen.1001130.s006.doc]

|  | **Beecham et al. Controls** | | | | | **New AD Controls** | | | | | **Udall Controls** | | | | |  |
| --- | --- | --- | --- | --- | --- | --- | --- | --- | --- | --- | --- | --- | --- | --- | --- | --- |
|  |  | **Genotype counts (freq.)*** | | |  |  | **Genotype counts (freq.)*** | | |  |  | **Genotype counts (freq.)*** | | |  |  |
| **SNP** | **Minor/Major**  **Allele (1/2)** | **1/1** | **1/2** | **1/3** | **HWE P**** | **Minor/Major**  **Allele (1/2)** | **1/1** | **1/2** | **1/3** | **HWE P**** | **Minor/Major**  **Allele (1/2)** | **1/1** | **1/2** | **1/3** | **HWEP**** | **FET P***** |
| rs2075650 | G/A | 11 (2.2%) | 106 (21.4%) | 379 (76.4%) | 0.31 | G/A | 10 (2.3%) | 85 (19.5%) | 342 (78.3%) | 0.11 | G/A | 3 (1.7%) | 50 (29.1%) | 119 (69.2%) | 0.58 | 0.15 |
| rs405509 | C/A | 150 (30.2%) | 238 (48%) | 108 (21.8%) | 0.47 | C/A | 121 (27.7%) | 221 (50.6%) | 95 (21.7%) | 0.77 | A/C | 42 (24.4%) | 87 (50.6%) | 43 (25%) | 1.00 | 0.69 |
| rs8106922 | G/A | 90 (18.2%) | 239 (48.3%) | 166 (33.5%) | 0.85 | G/A | 63 (14.4%) | 227 (51.9%) | 147 (33.6%) | 0.11 | G/A | 29 (16.9%) | 73 (42.4%) | 70 (40.7%) | 0.20 | 0.16 |
| rs157580 | G/A | 75 (15.1%) | 226 (45.6%) | 195 (39.3%) | 0.5 | G/A | 66 (15.1%) | 211 (48.3%) | 160 (36.6%) | 0.84 | G/A | 30 (17.4%) | 78 (45.3%) | 64 (37.2%) | 0.53 | 0.84 |
| rs439401 | A/G | 75 (15.1%) | 222 (44.8%) | 199 (40.1%) | 0.34 | A/G | 67 (15.4%) | 205 (47%) | 164 (37.6%) | 0.84 | A/G | 15 (8.7%) | 92 (53.5%) | 65 (37.8%) | 0.03 | 0.13 |
| rs11754661 | A/G | 1 (0.2%) | 51 (10.3%) | 443 (89.5%) | 1.00 | A/G | 1 (0.2%) | 41 (9.4%) | 394 (90.4%) | 1.00 | A/G | 0 (0%) | 18 (10.5%) | 154 (89.5%) | 1.00 | 0.95 |
| rs6859 | A/G | 89 (17.9%) | 256 (51.6%) | 151 (30.4%) | 0.32 | A/G | 78 (17.9%) | 207 (47.5%) | 151 (34.6%) | 0.62 | A/G | 28 (16.3%) | 78 (45.3%) | 66 (38.4%) | 0.53 | 0.36 |
| rs10402271 | C/A | 57 (11.5%) | 214 (43.1%) | 225 (45.4%) | 0.61 | C/A | 43 (9.8%) | 186 (42.6%) | 208 (47.6%) | 0.91 | C/A | 15 (8.7%) | 81 (47.1%) | 76 (44.2%) | 0.38 | 0.72 |
| rs6509916 | G/A | 99 (20%) | 234 (47.2%) | 163 (32.9%) | 0.36 | G/A | 85 (19.5%) | 211 (48.5%) | 139 (32%) | 0.77 | G/A | 32 (18.6%) | 85 (49.4%) | 55 (32%) | 1.00 | 0.99 |
| rs509512 | C/A | 107 (21.6%) | 241 (48.6%) | 148 (29.8%) | 0.65 | C/A | 93 (21.3%) | 232 (53.2%) | 111 (25.5%) | 0.18 | C/A | 34 (19.8%) | 91 (52.9%) | 47 (27.3%) | 0.45 | 0.58 |
| rs679670 | G/A | 89 (17.9%) | 225 (45.4%) | 182 (36.7%) | 0.19 | G/A | 70 (16%) | 204 (46.7%) | 163 (37.3%) | 0.69 | G/A | 25 (14.5%) | 89 (51.7%) | 58 (33.7%) | 0.43 | 0.63 |

* freq. = frequency (percent)

** P-value for deviation from Hardy-Weinberg Equilibrium (HWE)

*** P-value for Fisher’s Exact Test (FET)
