# Supplementary material for: Dementia Revealed: Novel Chromosome 6 Locus for Late-Onset Alzheimer Disease Provides Genetic Evidence for Folate-Pathway Abnormalities
Source: PLoS Genet. 2010 Sep 23;6(9):e1001130. doi: 10.1371/journal.pgen.1001130 (PMC2944795; doi:10.1371/journal.pgen.1001130)
Supplement: Table S5 — Demographic characteristics of participants, subsetted by study center, autopsy or clinical confirmation of case or control status, and by genotyping platform (mean ± SD or number (percent)). (0.07 MB DOC) [file pgen.1001130.s007.doc]

|  | **HIHG** | **CHGR** | **MSBB** | **Autopsy-Confirmed** | **Clinically-Confirmed** |
| --- | --- | --- | --- | --- | --- |
| **Number of subjects** | 1,024 | 663 | 349 | 349 | 1,687 |
| Cases (%) | 328 (32.0%) | 328 (49.5%) | 276 (79.1%) | 276 (79.1%) | 656 (38.9%) |
| **Cases** |  |  |  |  |  |
| Females (%) | 210 (64.0%) | 200 (61.0%) | 191 (69.2%) | 191 (69.2%) | 410 (62.5%) |
| Age-at-onset (yr) | 72.8 ± 7.1 | 73.4 ± 6.5 | 79.2 ± 10.6 | 79.2 ± 10.6 | 73.1 ± 6.9 |
| 0 copies of APOE ε4 (%) | 112 (34.2%) | 127 (38.7%) | 160 (58.0%) | 160 (58.0%) | 239 (36.4%) |
| 1 copy of APOE ε4 (%) | 147 (44.8%) | 155 (47.3%) | 96 (34.8%) | 96 (34.8%) | 302 (46.0%) |
| 2 copies of APOE ε4 (%) | 65 (19.8%) | 43 (13.1%) | 19 (6.9%) | 19 (6.9%) | 108 (16.5%) |
| Carrier status missing | 4 (1.2%) | 3 (0.9%) | 1 (0.4%) | 1 (0.4%) | 7 (1.1%) |
| **Controls** |  |  |  |  |  |
| Females (%) | 449 (64.5%) | 189 (56.4%) | 45 (61.6%) | 45 (61.6%) | 683 (61.9%) |
| Age-at-exam (yr) | 73.2 ± 7.3 | 73.1 ± 7.0 | 82.7 ± 9.5 | 82.7 ± 9.5 | 73.1 ± 7.2 |
| 0 copies of APOE ε4 (%) | 515 (74.0%) | 247 (73.7%) | 62 (85.0%) | 62 (85.0%) | 762 (73.9%) |
| 1 copy of APOE ε4 (%) | 151 (21.7%) | 70 (20.9%) | 10 (13.7%) | 10 (13.7%) | 221 (21.4%) |
| 2 copies of APOE ε4 (%) | 8 (1.2%) | 9 (2.7%) | 0 (0%) | 0 (0%) | 17 (1.7%) |
| Carrier status missing | 22 (3.2%) | 9 (2.7%) | 1 (1.4%) | 1 (1.4%) | 31 (3.0%) |

|  | **Illumina 550K** | **Illumina 610Quad** | **Illumina 1M** |
| --- | --- | --- | --- |
| **Number of subjects** | 2,036 | 172 | 877 |
| Cases (%) | 492 (49.9%) | 0 (0%) | 440 (50.2%) |
| **Cases** |  |  |  |
| Females (%) | 312 (63.4%) | -- | 289 (65.7%) |
| Age-at-onset (yr) | 72.9 ± 6.5 | -- | 76.5 ± 9.7 |
| 0 copies of APOE ε4 (%) | 169 (34.4%) | -- | 230 (52.3%) |
| 1 copy of APOE ε4 (%) | 234 (47.6%) | -- | 164 (37.3%) |
| 2 copies of APOE ε4 (%) | 86 (17.5%) | -- | 41 (9.3%) |
| carrier status missing | 3 (0.6%) | -- | 5 (1.1%) |
| **Controls** |  |  |  |
| Females (%) | 304 (61.4%) | 119 (69.2%) | 260 (59.5%) |
| Age-at-exam (yr) | 74.2 ± 6.5 | 71.4 ± 7.6 | 74.4 ± 9.1 |
| 0 copies of APOE ε4 (%) | 377 (76.2%) | 114 (66.3%) | 333 (76.2%) |
| 1 copy of APOE ε4 (%) | 105 (21.2%) | 50 (29.1%) | 76 (17.4%) |
| 2 copies of APOE ε4 (%) | 10 (2.0%) | 3 (1.7%) | 4 (0.9%) |
| carrier status missing | 3 (0.6%) | 5 (2.9%) | 24 (5.5%) |
