# Supplementary material for: Dementia Revealed: Novel Chromosome 6 Locus for Late-Onset Alzheimer Disease Provides Genetic Evidence for Folate-Pathway Abnormalities
Source: PLoS Genet. 2010 Sep 23;6(9):e1001130. doi: 10.1371/journal.pgen.1001130 (PMC2944795; doi:10.1371/journal.pgen.1001130)
Supplement: Table S6 — Changes in effect size and p-value with additional covariate adjustment for age, sex, and presence/absence of the APOE ε4 allele for SNP associations demonstrating P<10−5 in preliminary analyses of late-onset Alzheimer Disease. SNPs demonstrating association with late-onset Alzheimer Disease at P<10−5 as identified in Table 2, here showing results from logistic regression modeling with (1) no additional covariate adjustment, (2) additional covariate adjustment for age-at-onset (years, in cases only) and age-at-exam (years, in controls only) and sex, and (3) additional covariate adjustment for age-at-onset (years, in cases only) and age-at-exam (years, in controls only); sex; and presence presence/absence of the APOE ε4 allele. All models include, at minimum, covariate adjustment for principal components capturing population substructure. (0.04 MB DOC) [file pgen.1001130.s008.doc]

|  |  |  |  |  |  | **(1) Unadjusted** | | **(2) Adjusted for Age* and Sex** | | **(3) Adjusted for Age*, Sex, & +/- APOE ε4** | |
| --- | --- | --- | --- | --- | --- | --- | --- | --- | --- | --- | --- |
| **SNP** | **Chr** | **Location** | **Gene***** | **Function***** | **Minor Allele (Freq.)** | **OR* (95% CI**)** | **P** | **OR* (95% CI**)** | **P** | **OR* (95% CI**)** | **P** |
| rs2075650 | 19 | 50087459 | *TOMM40* | intron | G (0.2) | 2.96 (2.50, 3.50) | 1.30×10^-36 | 3.08 (2.59, 3.67) | 3.72×10^-36 | 1.47 (1.17, 1.86) | 0.00104 |
| rs405509 | 19 | 50100676 | *APOE* |  | C (0.48) | 0.62 (0.55, 0.70) | 1.47×10^-13 | 0.61 (0.54, 0.70) | 3.55×10^-13 | 0.79 (0.69, 0.92) | 0.002 |
| rs8106922 | 19 | 50093506 | *TOMM40* | intron | G (0.36) | 0.62 (0.54, 0.71) | 3.10×10^-12 | 0.62 (0.54, 0.72) | 2.93×10^-11 | 0.93 (0.80, 1.10) | 0.405 |
| rs157580 | 19 | 50087106 | *TOMM40* | intron | G (0.35) | 0.66 (0.57, 0.75) | 1.22×10^-9 | 0.65 (0.56, 0.74) | 1.39×10^-9 | 0.94 (0.80, 1.10) | 0.457 |
| rs439401 | 19 | 50106291 | *LOC100129500* | intron | A (0.34) | 0.66 (0.57, 0.75) | 1.76×10^-9 | 0.66 (0.57, 0.76) | 5.60×10^-9 | 0.99 (0.84, 1.17) | 0.914 |
| rs11754661 | 6 | 151248771 | *MTHFD1L* | intron | A (0.07) | 2.03 (1.58, 2.62) | 4.70×10^-8 | 2.03 (1.56, 2.64) | 1.42×10^-7 | 2.01 (1.51, 2.67) | 1.64×10^-6 |
| rs6859 | 19 | 50073874 | *PVRL2* | intron | A (0.46) | 1.41 (1.24, 1.60) | 1.06×10^-7 | 1.42 (1.24, 1.61) | 1.93×10^-7 | 1.11 (0.96, 1.28) | 0.163 |
| rs10402271 | 19 | 50021054 |  |  | C (0.36) | 1.39 (1.22, 1.59) | 7.26×10^-7 | 1.45 (1.26, 1.66) | 9.82×10^-8 | 1.13 (0.97, 1.31) | 0.114 |
| rs6509916 | 19 | 60254214 | *RDH13* | intron | G (0.46) | 1.34 (1.18, 1.52) | 5.83×10^-6 | 1.35 (1.18, 1.54) | 8.32×10^-6 | 1.33 (1.16, 1.54) | 8.11×10^-5 |
| rs509512 | 11 | 105350133 | *GRIA4* | intron | C (0.43) | 0.75 (0.66, 0.85) | 7.37×10^-6 | 0.74 (0.64, 0.84) | 5.64×10^-6 | 0.77 (0.67, 0.89) | 0.000341 |
| rs679670 | 6 | 138179244 |  |  | G (0.37) | 0.74 (0.65, 0.85) | 9.83×10^-6 | 0.75 (0.65, 0.86) | 3.54×10^-5 | 0.73 (0.63, 0.85) | 3.57×10^-5 |

* OR = Odds Ratio

** CI = Confidence Interval

*** Gene Annotation using SNPper database (Riva and Kohane, 2002) [1]
