# Supplementary material for: Dementia Revealed: Novel Chromosome 6 Locus for Late-Onset Alzheimer Disease Provides Genetic Evidence for Folate-Pathway Abnormalities
Source: PLoS Genet. 2010 Sep 23;6(9):e1001130. doi: 10.1371/journal.pgen.1001130 (PMC2944795; doi:10.1371/journal.pgen.1001130)
Supplement: Table S7 — Associations with late-onset Alzheimer Disease of MTHFD1L haplotypes incorporating SNP rs11754661, with adjustment for covariates from principal components capturing population substructure, evaluated in the Discovery GWAS dataset of 931 independent cases and 1,104 independent cognitively normal controls. (0.03 MB DOC) [file pgen.1001130.s009.doc]

|  | **Strongest Haplotypic Association** | | | | **Second Strongest Haplotypic Association** | | | |
| --- | --- | --- | --- | --- | --- | --- | --- | --- |
| **Haplotype** | **Allele** | **F*** | **OR**** | **P** | **Allele** | **F*** | **OR**** | **P** |
| Haplotype 1^a^ | AAA | 0.0696 | 2.03 | 4.60×10^-8 | AGA | 0.654 | 0.851 | 0.0157 |
| Haplotype 2^b^ | GG | 0.2 | 1.32 | 0.00054 | AA | 0.55 | 0.882 | 0.05 |
| Extended Haplotype 1^c^ | GGAAA | 0.0629 | 2.09 | 6.54×10^-8 | AAAGA | 0.529 | 0.871 | 0.0325 |
| Extended Haplotype 2^d^ | GGGAAGAGAAGGAAAG | 0.0115 | 3.51 | 0.000206 | GGAAGAAGGAGGAAAG | 0.0218 | 2.3 | 0.0003 |

^a^ Haplotype 1: rs2073066- rs11754661- rs13201018

^b^ Haplotype 2: rs2839947- rs11757561

^c^ Extended Haplotype 1: rs2839947- rs11757561- rs2073066- rs11754661- rs13201018

^d^ Extended Haplotype 2: rs17348429- rs17426727- rs803410- rs6917461- rs803407- rs803403- rs17348890- rs17427389- rs9397027- rs10484779- rs2839947- rs11757561- rs2073066- rs11754661- rs13201018- rs17349743

* Freq. = Frequency

** OR = Odds Ratio
