# Supplementary material for: Dementia Revealed: Novel Chromosome 6 Locus for Late-Onset Alzheimer Disease Provides Genetic Evidence for Folate-Pathway Abnormalities
Source: PLoS Genet. 2010 Sep 23;6(9):e1001130. doi: 10.1371/journal.pgen.1001130 (PMC2944795; doi:10.1371/journal.pgen.1001130)
Supplement: Table S8 — Genotyping or imputation of SNPs associated with LOAD at P<10−4. Index indicating whether single nucleotide polymorphisms (SNPs) demonstrating association with late-onset Alzheimer Disease at P<10−4 in association tests adjusting for population substructure in the Discovery dataset where genotyped or imputed in the Discovery dataset (931 independent cases and 1,104 independent cognitively normal controls) or any of the Replication datasets, including the from the Alzheimer's Disease Neuroimaging Initiative (ADNI) [60] (147 cases and 182 controls), the Framingham Study SHARe dataset (SHARe) [61] (86 cases and 1,200 controls (all unrelated)), the Reiman, et al., LOAD GWAS dataset (TGEN) [15] (859 cases and 552 controls), and an additional set of LOAD cases and controls independent of the Discovery dataset and not used in prior publications (ADRC) [51] (246 LOAD cases and 69 cognitively normal controls). (0.24 MB DOC) [file pgen.1001130.s010.doc]

|  |  |  |  | **Replication GWAS** | | | |
| --- | --- | --- | --- | --- | --- | --- | --- |
| **SNP** | **Chr** | **Location** | **Discover GWAS** | **ADRC** | **ADNI** | **SHARe** | **TGEN** |
| rs2075650 | 19 | 50087459 | Genotyped | Imputed | Genotyped | Imputed | Imputed |
| rs157582 | 19 | 50088059 | Genotyped | Imputed | Imputed | Imputed | Imputed |
| rs405509 | 19 | 50100676 | Genotyped | Imputed | Genotyped | Imputed | Imputed |
| rs8106922 | 19 | 50093506 | Genotyped | Imputed | Genotyped | Imputed | Imputed |
| rs157580 | 19 | 50087106 | Genotyped | Imputed | Genotyped | Imputed | Imputed |
| rs439401 | 19 | 50106291 | Genotyped | Imputed | Genotyped | Imputed | Imputed |
| rs11754661 | 6 | 151248771 | Genotyped | Imputed | Genotyped | Imputed | Imputed |
| rs6859 | 19 | 50073874 | Genotyped | Imputed | Genotyped | Imputed | Imputed |
| rs10402271 | 19 | 50021054 | Genotyped | Genotyped | Genotyped | Genotyped | Genotyped |
| rs2252811 | 10 | 49862126 | Genotyped | Imputed | Imputed | Imputed | Imputed |
| rs6773562 | 3 | 23633034 | Genotyped | Genotyped | Imputed | Genotyped | Genotyped |
| rs7699794 | 4 | 138565789 | Genotyped | Imputed | Imputed | Imputed | Imputed |
| rs10005776 | 4 | 111235463 | Genotyped | Imputed | Imputed | Imputed | Imputed |
| rs12645160 | 4 | 160688870 | Genotyped | Imputed | Genotyped | Imputed | Imputed |
| rs12047155 | 1 | 50189517 | Genotyped | Imputed | Imputed | Imputed | Imputed |
| rs6509916 | 19 | 60254214 | Genotyped | Imputed | Genotyped | Imputed | Imputed |
| rs509512 | 11 | 105350133 | Genotyped | Imputed | Genotyped | Imputed | Imputed |
| rs679670 | 6 | 138179244 | Genotyped | Imputed | Genotyped | Imputed | Imputed |
| rs9660278 | 1 | 50272772 | Genotyped | Imputed | Imputed | Imputed | Imputed |
| rs4926825 | 1 | 49831163 | Genotyped | Imputed | Imputed | Imputed | Imputed |
| rs1244096 | 12 | 123481044 | Genotyped | Imputed | Genotyped | Imputed | Imputed |
| rs799419 | 6 | 138180548 | Genotyped | Imputed | Genotyped | Imputed | Imputed |
| rs669397 | 11 | 105351597 | Genotyped | Imputed | Genotyped | Genotyped | Genotyped |
| rs10225470 | 7 | 54155997 | Genotyped | Imputed | Genotyped | Imputed | Imputed |
| rs8074294 | 17 | 61902137 | Genotyped | Imputed | Genotyped | Imputed | Imputed |
| rs1167272 | 1 | 49658574 | Genotyped | Imputed | Imputed | Imputed | Imputed |
| rs17379721 | 1 | 50049596 | Genotyped | Imputed | Imputed | Imputed | Imputed |
| rs11025237 | 11 | 19726288 | Genotyped | Genotyped | Imputed | Imputed | Imputed |
| rs7091819 | 10 | 26028836 | Genotyped | Genotyped | Genotyped | Genotyped | Genotyped |
| rs12083887 | 1 | 118683212 | Genotyped | Imputed | Imputed | Imputed | Imputed |
| rs8113032 | 19 | 60245950 | Genotyped | Genotyped | Genotyped | Genotyped | Genotyped |
| rs12135821 | 1 | 118744972 | Genotyped | Imputed | Imputed | Imputed | Imputed |
| rs11131099 | 3 | 823802 | Genotyped | Genotyped | Imputed | Genotyped | Genotyped |
| rs1893953 | 4 | 160693858 | Genotyped | Imputed | Imputed | Imputed | Imputed |
| rs4676049 | 2 | 109001689 | Genotyped | Imputed | Genotyped | Imputed | Imputed |
| rs7303876 | 12 | 58421479 | Genotyped | Imputed | Genotyped | Imputed | Imputed |
| rs1856297 | 1 | 49810849 | Genotyped | Imputed | Imputed | Imputed | Imputed |
| rs4388744 | 1 | 50294191 | Genotyped | Imputed | Imputed | Imputed | Imputed |
| rs11100238 | 4 | 160696507 | Genotyped | Imputed | Imputed | Imputed | Imputed |
| rs4489606 | 1 | 50287140 | Genotyped | Imputed | Imputed | Imputed | Imputed |
| rs12255607 | 10 | 20181408 | Genotyped | Imputed | Imputed | Imputed | Imputed |
| rs4926814 | 1 | 49729723 | Genotyped | Imputed | Imputed | Imputed | Imputed |
| rs4926547 | 1 | 50319397 | Genotyped | Genotyped | Imputed | Genotyped | Genotyped |
| rs11038913 | 11 | 46516306 | Genotyped | Imputed | Imputed | Imputed | Imputed |
| rs1415985 | 1 | 49703336 | Genotyped | Imputed | Imputed | Imputed | Imputed |
| rs2975139 | 12 | 16393084 | Genotyped | Imputed | Genotyped | Imputed | Imputed |
| rs17034806 | 2 | 109002337 | Genotyped | Genotyped | Genotyped | Imputed | Imputed |
| rs2305543 | 19 | 60251527 | Genotyped | Imputed | Imputed | Imputed | Imputed |
| rs11720720 | 3 | 65794510 | Genotyped | Imputed | Imputed | Imputed | Imputed |
| rs2529491 | 7 | 110959870 | Genotyped | Imputed | Imputed | Imputed | Imputed |
| rs1360873 | 13 | 63489710 | Genotyped | Imputed | Genotyped | Imputed | Imputed |
| rs6957883 | 7 | 147870112 | Genotyped | Imputed | Imputed | Imputed | Imputed |
| rs1538981 | 10 | 31451361 | Genotyped | Imputed | Imputed | Imputed | Imputed |
| rs1185222 | 1 | 49731548 | Genotyped | Imputed | Imputed | Imputed | Imputed |
| rs1727987 | 1 | 49761808 | Genotyped | Imputed | Imputed | Imputed | Imputed |
| rs10888665 | 1 | 49911493 | Genotyped | Imputed | Imputed | Imputed | Imputed |
| rs2050876 | 10 | 31093734 | Genotyped | Genotyped | Genotyped | Imputed | Genotyped |
| rs1167262 | 1 | 49646567 | Genotyped | Imputed | Imputed | Imputed | Imputed |
| rs10888679 | 1 | 50338853 | Genotyped | Imputed | Genotyped | Imputed | Imputed |
| rs1891667 | 1 | 49867972 | Genotyped | Genotyped | Imputed | Genotyped | Genotyped |
| rs2000886 | 4 | 160740930 | Genotyped | Imputed | Imputed | Imputed | Imputed |
| rs1112687 | 1 | 49841291 | Genotyped | Imputed | Imputed | Imputed | Imputed |
| rs7019702 | 9 | 132897543 | Genotyped | Genotyped | Imputed | Imputed | Imputed |
| rs3957 | 1 | 49630498 | Genotyped | Imputed | Imputed | Imputed | Imputed |
| rs9989761 | 2 | 132855872 | Genotyped | Imputed | Genotyped | Imputed | Imputed |
| rs12049328 | 1 | 49463210 | Genotyped | Imputed | Imputed | Imputed | Imputed |
| rs1343161 | 1 | 49883437 | Genotyped | Imputed | Imputed | Genotyped | Genotyped |
| rs16974980 | 16 | 83530064 | Genotyped | Imputed | Genotyped | Imputed | Imputed |
| rs13151952 | 4 | 138539922 | Genotyped | Imputed | Imputed | Imputed | Imputed |
| rs1713417 | 14 | 19933651 | Genotyped | Genotyped | Imputed | Genotyped | Genotyped |
| rs6693294 | 1 | 49651709 | Genotyped | Imputed | Imputed | Imputed | Imputed |
| rs4820297 | 22 | 36435927 | Genotyped | Imputed | Genotyped | Imputed | Imputed |
| rs1577969 | 1 | 49637882 | Genotyped | Imputed | Imputed | Imputed | Imputed |
| rs10736388 | 1 | 50108115 | Genotyped | Imputed | Imputed | Genotyped | Genotyped |
| rs1112368 | 1 | 49932177 | Genotyped | Imputed | Imputed | Imputed | Imputed |
| rs7530169 | 1 | 49941178 | Genotyped | Imputed | Imputed | Imputed | Imputed |
| rs5930403 | 23 | 129318208 | Genotyped | Imputed | Genotyped | Imputed | Imputed |
| rs1179484 | 1 | 49681231 | Genotyped | Genotyped | Imputed | Genotyped | Genotyped |
| rs1167270 | 1 | 49685647 | Genotyped | Imputed | Imputed | Imputed | Imputed |
| rs5977248 | 23 | 129329168 | Genotyped | Genotyped | Genotyped | Imputed | Imputed |
| rs1494462 | 1 | 49568744 | Genotyped | Imputed | Imputed | Imputed | Imputed |
| rs2301343 | 2 | 40533653 | Genotyped | Imputed | Genotyped | Imputed | Imputed |
| rs2064179 | 23 | 129236874 | Genotyped | Imputed | Genotyped | Imputed | Imputed |
| rs2846215 | 11 | 105376028 | Genotyped | Imputed | Imputed | Imputed | Imputed |
| rs4926812 | 1 | 49586400 | Genotyped | Imputed | Imputed | Imputed | Imputed |
| rs1338214 | 1 | 49596084 | Genotyped | Genotyped | Imputed | Genotyped | Genotyped |
| rs10259067 | 7 | 95069392 | Genotyped | Imputed | Imputed | Imputed | Imputed |
| rs10244338 | 7 | 70359758 | Genotyped | Imputed | Genotyped | Imputed | Imputed |
| rs6588362 | 1 | 50066939 | Genotyped | Imputed | Imputed | Imputed | Imputed |
| rs10888669 | 1 | 50022860 | Genotyped | Genotyped | Imputed | Genotyped | Genotyped |
| rs2787693 | 1 | 49688435 | Genotyped | Imputed | Imputed | Imputed | Imputed |
| rs589104 | 11 | 105312982 | Genotyped | Imputed | Genotyped | Imputed | Imputed |
| rs3092217 | 20 | 39813482 | Genotyped | Imputed | Imputed | Imputed | Imputed |
| rs4926545 | 1 | 50152063 | Genotyped | Imputed | Imputed | Imputed | Imputed |
| rs6697839 | 1 | 50163925 | Genotyped | Imputed | Imputed | Imputed | Imputed |
| rs10788924 | 1 | 50168618 | Genotyped | Imputed | Imputed | Imputed | Imputed |
| rs11603669 | 11 | 134212161 | Genotyped | Genotyped | Imputed | Genotyped | Genotyped |
| rs5932752 | 23 | 129334460 | Genotyped | Imputed | Genotyped | Imputed | Imputed |
| rs12036551 | 1 | 49555928 | Genotyped | Imputed | Imputed | Imputed | Imputed |
| rs7795083 | 7 | 70376454 | Genotyped | Genotyped | Imputed | Imputed | Imputed |
| rs3798267 | 6 | 46058786 | Genotyped | Imputed | Genotyped | Imputed | Imputed |
| rs6029791 | 20 | 39794198 | Genotyped | Imputed | Imputed | Imputed | Imputed |
| rs10424969 | 19 | 60258324 | Genotyped | Genotyped | Imputed | Genotyped | Genotyped |
| rs6693846 | 1 | 50087939 | Genotyped | Imputed | Imputed | Imputed | Imputed |
| rs7544728 | 1 | 50112236 | Genotyped | Imputed | Imputed | Imputed | Imputed |
| rs2832594 | 21 | 30388188 | Genotyped | Imputed | Imputed | Genotyped | Imputed |
| rs1939153 | 11 | 105256936 | Genotyped | Imputed | Genotyped | Imputed | Imputed |
| rs11842468 | 13 | 55833285 | Genotyped | Imputed | Genotyped | Imputed | Imputed |
| rs12713404 | 2 | 59860209 | Genotyped | Imputed | Imputed | Imputed | Imputed |
| rs12743369 | 1 | 50337003 | Genotyped | Imputed | Imputed | Imputed | Imputed |
| rs256335 | 19 | 39007736 | Genotyped | Imputed | Genotyped | Imputed | Imputed |
| rs7540194 | 1 | 49563736 | Genotyped | Imputed | Imputed | Imputed | Imputed |
| rs2714068 | 11 | 122904751 | Genotyped | Imputed | Genotyped | Imputed | Imputed |
| rs4806636 | 19 | 60240370 | Genotyped | Imputed | Imputed | Imputed | Imputed |
| rs3899856 | 1 | 50016441 | Genotyped | Imputed | Imputed | Imputed | Imputed |
| rs6695041 | 1 | 50018096 | Genotyped | Imputed | Imputed | Imputed | Imputed |
| rs5932738 | 23 | 129242314 | Genotyped | Imputed | Imputed | Imputed | Imputed |
| rs1654431 | 19 | 60241570 | Genotyped | Imputed | Imputed | Imputed | Imputed |
| rs241472 | 1 | 49544440 | Genotyped | Genotyped | Imputed | Imputed | Genotyped |
| rs11061995 | 12 | 1815303 | Genotyped | Imputed | Genotyped | Imputed | Imputed |
| rs2529489 | 7 | 110947132 | Genotyped | Genotyped | Genotyped | Genotyped | Genotyped |
| rs12976416 | 19 | 34053326 | Genotyped | Imputed | Imputed | Imputed | Imputed |
| rs1010978 | 23 | 129307141 | Genotyped | Imputed | Imputed | Imputed | Imputed |
| rs6449493 | 5 | 60082253 | Genotyped | Imputed | Imputed | Imputed | Imputed |
| rs4830186 | 23 | 129340004 | Genotyped | Imputed | Imputed | Imputed | Imputed |
| rs4830187 | 23 | 129340424 | Genotyped | Genotyped | Imputed | Imputed | Imputed |
| rs4582848 | 1 | 50084787 | Genotyped | Genotyped | Imputed | Genotyped | Genotyped |
| rs4779542 | 15 | 29639276 | Genotyped | Imputed | Imputed | Imputed | Imputed |
| rs7574523 | 2 | 132858237 | Genotyped | Imputed | Imputed | Imputed | Imputed |
| rs869058 | 6 | 90312689 | Genotyped | Imputed | Imputed | Imputed | Imputed |
| rs4234232 | 3 | 34051850 | Genotyped | Imputed | Genotyped | Imputed | Imputed |
| rs10912537 | 1 | 169433156 | Genotyped | Imputed | Genotyped | Imputed | Imputed |
| rs1800291 | 23 | 153811479 | Genotyped | Imputed | Imputed | Imputed | Imputed |
| rs3777599 | 6 | 46050909 | Genotyped | Imputed | Imputed | Imputed | Imputed |
| rs1393576 | 4 | 160707662 | Genotyped | Imputed | Genotyped | Genotyped | Genotyped |
| rs5932754 | 23 | 129342752 | Genotyped | Genotyped | Imputed | Imputed | Imputed |
| rs13427042 | 2 | 132858435 | Genotyped | Imputed | Imputed | Imputed | Imputed |
| rs10282292 | 7 | 110879714 | Genotyped | Imputed | Genotyped | Imputed | Imputed |
| rs7080202 | 10 | 31097100 | Genotyped | Imputed | Imputed | Imputed | Imputed |
| rs1331501 | 9 | 92432152 | Genotyped | Genotyped | Genotyped | Genotyped | Genotyped |
| rs867991 | 10 | 31429271 | Genotyped | Imputed | Imputed | Imputed | Imputed |
| rs2292585 | 10 | 49856323 | Genotyped | Imputed | Imputed | Imputed | Imputed |
| rs4830188 | 23 | 129342104 | Genotyped | Genotyped | Imputed | Imputed | Imputed |
| rs1998587 | 9 | 78268523 | Genotyped | Genotyped | Imputed | Genotyped | Genotyped |
| rs9544105 | 13 | 75456154 | Genotyped | Imputed | Genotyped | Imputed | Imputed |
| rs12610605 | 19 | 50062678 | Genotyped | Genotyped | Genotyped | Genotyped | Imputed |
