# Supplementary material for: Dementia Revealed: Novel Chromosome 6 Locus for Late-Onset Alzheimer Disease Provides Genetic Evidence for Folate-Pathway Abnormalities
Source: PLoS Genet. 2010 Sep 23;6(9):e1001130. doi: 10.1371/journal.pgen.1001130 (PMC2944795; doi:10.1371/journal.pgen.1001130)
Supplement: Text S1 — Supplementary methods describing haplotype analyses. (0.02 MB DOC) [file pgen.1001130.s011.doc]

**Supplementary Methods**

**Haplotype Analyses**

In order to define haplotypes, Linkage Disequilibrium (LD) structure in the vicinity of SNP rs11754661 was examined with the Haploview program [3] estimating both *D’* and *r^2^* measures for this region. Haplotypes were constructed using LD blocks assigned by Haploview, including the LD block containing the SNP (consisting of rs2073066, rs11754661, and rs13201018; analyzed as “Haplotype 1”) and immediately adjacent to the SNP (consisting of rs2839947 and rs11757561; analyzed as “Haplotype 2”) were examined for association. Extended haplotypes were constructed by further incorporating SNPs from LD Blocks immediately adjacent to the Haplotype 1 block in MTHFD1L, which included the Haplotype 2 block and a third, larger block incorporating SNPs rs17348429, rs17426727, rs803410, rs6917461, rs803407, rs803403, rs17348890, rs17427389, rs9397027, and rs10484779 (labeled here as “Haplotype 3”). This set of haplotypes included “Extended Haploype 1” (comprising SNPs from Haplotype 1 and 2 blocks), and “Extended Haplotype 2” (comprising SNPs from Haplotype 1, 2, and 3 blocks).

Haplotypic association tests were performed in a manner similar to genotypic association analysis, using a logistic regression approach with covariate adjusment for loadings taken from the first three principal components identified in EIGENSTRAT [4] to account for population substructure. All analyses were performed using the “--hap-logistic” function in the PLINK software package [5].

**REFERENCES**

1. Riva A, Kohane IS (2002) SNPper: retrieval and analysis of human SNPs. Bioinformatics 18: 1681-1685.

2. Beecham GW, Martin ER, Li YJ, Slifer MA, Gilbert JR, et al. (2009) Genome-wide association study implicates a chromosome 12 risk locus for late-onset Alzheimer disease. Am J Hum Genet 84: 35-43.

3. Barrett JC, Fry B, Maller J, Daly MJ (2005) Haploview: analysis and visualization of LD and haplotype maps. Bioinformatics 21: 263-265.

4. Price AL, Patterson NJ, Plenge RM, Weinblatt ME, Shadick NA, et al. (2006) Principal components analysis corrects for stratification in genome-wide association studies. Nat Genet 38: 904-909.

5. Purcell S, Neale B, Todd-Brown K, Thomas L, Ferreira MA, et al. (2007) PLINK: a tool set for whole-genome association and population-based linkage analyses. Am J Hum Genet 81: 559-575.
